# Predictive value for cardiovascular events of common carotid intima media thickness and its rate of change in individuals at high cardiovascular risk – Results from the PROG-IMT collaboration

**DOI:** 10.1371/journal.pone.0191172

**Published:** 2018-04-12

**Authors:** Matthias W. Lorenz, Lu Gao, Kathrin Ziegelbauer, Giuseppe Danilo Norata, Jean Philippe Empana, Irene Schmidtmann, Hung-Ju Lin, Stela McLachlan, Lena Bokemark, Kimmo Ronkainen, Mauro Amato, Ulf Schminke, Sathanur R. Srinivasan, Lars Lind, Shuhei Okazaki, Coen D. A. Stehouwer, Peter Willeit, Joseph F. Polak, Helmuth Steinmetz, Dirk Sander, Holger Poppert, Moise Desvarieux, M. Arfan Ikram, Stein Harald Johnsen, Daniel Staub, Cesare R. Sirtori, Bernhard Iglseder, Oscar Beloqui, Gunnar Engström, Alfonso Friera, Francesco Rozza, Wuxiang Xie, Grace Parraga, Liliana Grigore, Matthieu Plichart, Stefan Blankenberg, Ta-Chen Su, Caroline Schmidt, Tomi-Pekka Tuomainen, Fabrizio Veglia, Henry Völzke, Giel Nijpels, Johann Willeit, Ralph L. Sacco, Oscar H. Franco, Heiko Uthoff, Bo Hedblad, Carmen Suarez, Raffaele Izzo, Dong Zhao, Thapat Wannarong, Alberico Catapano, Pierre Ducimetiere, Christine Espinola-Klein, Kuo-Liong Chien, Jackie F. Price, Göran Bergström, Jussi Kauhanen, Elena Tremoli, Marcus Dörr, Gerald Berenson, Kazuo Kitagawa, Jacqueline M. Dekker, Stefan Kiechl, Matthias Sitzer, Horst Bickel, Tatjana Rundek, Albert Hofman, Ellisiv B. Mathiesen, Samuela Castelnuovo, Manuel F. Landecho, Maria Rosvall, Rafael Gabriel, Nicola de Luca, Jing Liu, Damiano Baldassarre, Maryam Kavousi, Eric de Groot, Michiel L. Bots, David N. Yanez, Simon G. Thompson, on behalf of the PROG-IMT study group

**Affiliations:** 1 Department of Neurology, Goethe University, Frankfurt am Main, Germany; 2 MRC Biostatistics Unit, Institute of Public Health, University Forvie Site, Cambridge, United Kingdom; 3 Department of Medical Biotechnology and Translational Medicine, Università degli Studi di Milano, Milano, Italy; 4 SISA Center for the Study of Atherosclerosis, Bassini Hospital, Cinisello Balsamo, Italy; 5 Paris Cardiovascular Research Centre (PARCC), University Paris Descartes, Sorbonne Paris Cité, UMR, Paris, France; 6 Institut fuer Medizinische Biometrie, Epidemiologie und Informatik (IMBEI), Universitaetsmedizin Mainz, Mainz, Germany; 7 Department of Internal Medicine, National Taiwan University Hospital, Taipei, Taiwan; 8 Usher Institute of Population Health Sciences and Informatics, University of Edinburgh, Edinburgh, United Kingdom; 9 Wallenberg Laboratory for Cardiovascular Research, Institution for Medicin, Department for Molecular and Clinical Medicine, Sahlgrenska Academy, Gothenburg University, Gothenburg, Sweden; 10 Institute of Public Health and Clinical Nutrition, University of Eastern Finland, Kuopio Campus, Kuopio, Finland; 11 Centro Cardiologico Monzino, IRCCS, Milano, Italy; 12 Department of Neurology, Greifswald University Clinic, Greifswald, Germany; 13 Center for Cardiovascular Health, Department of Epidemiology, Biochemistry, Tulane University School of Public Health and Tropical Medicine, New Orleans, Louisiana, United States of America; 14 Department of Medicine, Uppsala University, Uppsala, Sweden; 15 Department of Neurology, Osaka University Graduate School of Medicine, Osaka, Japan; 16 Department of Internal Medicine and Cardiovascular Research Institute Maastricht (CARIM), Maastricht University Medical Centre, Maastricht, the Netherlands; 17 Department of Neurology, Medical University Innsbruck, Innsbruck, Austria; 18 Department of Public Health and Primary Care, School of Clinical Medicine, University of Cambridge, Cambridge, United Kingdom; 19 Tufts University School of Medicine, Tufts Medical Center, Boston, Massachusetts, United States of America; 20 Department of Neurology, Benedictus Hospital Tutzing & Feldafing, Feldafing, Germany; 21 Department of Neurology, Technische Universität München, Munich, Germany; 22 Department of Epidemiology,Mailman School of Public Health,Columbia University, New York, United States of America; 23 Department of Epidemiology, Erasmus University Medical Center, Rotterdam, the Netherlands; 24 Department of Neurology, Erasmus University Medical Center, Rotterdam, the Netherlands; 25 Department of Radiology, Erasmus University Medical Center, Rotterdam, the Netherlands; 26 Department of Clinical Medicine, Uit The Arctic University of Norway, Tromsø, Norway; 27 Department of Neurology, University Hospital of Northern Norway, Tromsø, Norway; 28 Department of Angiology, University Hospital Basel, Basel, Switzerland; 29 Center of Dyslipidemias, Niguarda Ca’ Granda Hospital, Milano, Italy; 30 Parcelsus Medical University, Salzburg, Austria; 31 Department of Geriatric Medicine, Gemeinnützige Salzburger Landeskliniken Betriebsgesellschaft GmbH Christian-Doppler-Klinik, Salzburg, Austria; 32 Department of Internal Medicine, University Clinic of Navarra, Navarra, Spain; 33 Department of Clinical Sciences in Malmö, Lund University, Malmö, Sweden; 34 Radiology Department, Hospital Universitario de la Princesa, Universidad Autónoma de Madrid, Madrid, Spain; 35 School of Medicine, Federico II University, Naples, Italy; 36 Department of Epidemiology, Beijing Institute of Heart, Lung and Blood Vessel Diseases,Beijing Anzhen Hospital, Capital Medical University, Beijing, China; 37 Robarts Research Institute, Western University, London, Ontario, Canada; 38 Centro Sisa per lo Studio della Aterosclerosi, Bassini Hospital, Cinisello Balsamo, Italy; 39 Assistance Publique, Hôpitaux de Paris, Hôpital Broca, Paris, France; 40 2nd Department of Medicine, Johannes Gutenberg-Universität, Mainz, Germany; 41 Department of Cardiology, University Hospital Hamburg-Eppendorf, Hamburg, Germany; 42 Wallenberg Laboratory for Cardiovascular Research, University of Gothenburg, Gothenburg, Sweden; 43 German Center for Cardiovascular Research (DZHK),partner site Greifswald, Greifswald, Germany; 44 Institute for Community Medicine, SHIP/Clinical-Epidemiological Research, Greifswald, Germany; 45 Department of General Practice, VU University Medical Center, Amsterdam, the Netherlands; 46 EMGO Institute for Health and Care Research, VU University Medical Center, Amsterdam, the Netherlands; 47 Department of Neurology, Miller School of Medicine, University of Miami, Miami, Florida, United States of America; 48 Department of Epidemiology, Erasmus MC, University Medical Center Rotterdam, Rotterdam, the Netherlands; 49 Internal Medicine Department, Hospital Universitario de la Princesa, Universidad Autónoma de Madrid, Madrid, Spain; 50 Stroke Prevention & Atherosclerosis Research Centre, Robarts Research Institute, Western University, London, Ontario, Canada; 51 Department of Internal Medicine, Faculty of Medicine, Faculty of Medicine Siriraj Hospital, Mahidol University, Bangkok, Thailand; 52 IRCSS Multimedica, Milan, Italy; 53 Department of Pharmacological and Biomolecular Sciences, University of Milan, Milan, Italy; 54 University Paris_Sud Xi, Kremlin-Bicêtre, Le Kremlin-Bicêtre, France; 55 2nd Department of Medicine, Johannes-Gutenberg University, Mainz, Germany; 56 Institute of Epidemiology and Preventive Medicine, College of Public Health,National Taiwan University, Taipei, Taiwan; 57 Wallenberg Laboratory for Cardiovascular Research, Sahlgrenska Academy, Gothenburg University, Götheborg, Sweden; 58 Department B for Internal Medicine, University Medicine Greifswald, Greifswald, Germany; 59 Department of Medicine, Pediatrics, Biochemistry, Epidemiology, Tulane University School of Medicine and School of Public Health and Tropical Medicine, New Orleans, Louisiana, United States of America; 60 Department of Neurology, Tokyo Women's Medical University, Tokyo, Japan; 61 Department of Epidemiology and Biostatistics, EMGO Institute for Health and Care Research, VU University Medical Center, Amsterdam, the Netherlands; 62 Department of Neuology, Klinikum Herford, Herford, Germany; 63 Department of Psychiatry and Psychotherapy, Technische Universität München, Munich, Germany; 64 Department of Clinical Sciences in Malmö, Lund University, Malmö, Sweden; 65 Escuela National de Sanidad, Instituto de Salud Carlos III, Madrid, Spain; 66 Department of Epidemiology and Biostatistics, Erasmus Medical Center, Rotterdam, the Netherlands; 67 Imagelabonline & Cardiovascular, Eindhoven and Lunteren, the Netherlands; 68 Department of Clinical Epidemiology, Biostatistics and Bioinformatics, Academic Medical Centre, Amsterdam, the Netherlands; 69 Julius Center for Health Sciences and Primary Care, University Medical Center Utrecht, Utrecht, the Netherlands; 70 Department of Biostatistics, University of Washington, Seattle, Washington, United States of America; Universita degli Studi di Perugia, ITALY

## Abstract

**Aims:**

Carotid intima media thickness (CIMT) predicts cardiovascular (CVD) events, but the predictive value of CIMT change is debated. We assessed the relation between CIMT change and events in individuals at high cardiovascular risk.

**Methods and results:**

From 31 cohorts with two CIMT scans (total n = 89070) on average 3.6 years apart and clinical follow-up, subcohorts were drawn: (A) individuals with at least 3 cardiovascular risk factors without previous CVD events, (B) individuals with carotid plaques without previous CVD events, and (C) individuals with previous CVD events. Cox regression models were fit to estimate the hazard ratio (HR) of the combined endpoint (myocardial infarction, stroke or vascular death) per standard deviation (SD) of CIMT change, adjusted for CVD risk factors. These HRs were pooled across studies.

In groups A, B and C we observed 3483, 2845 and 1165 endpoint events, respectively. Average common CIMT was 0.79mm (SD 0.16mm), and annual common CIMT change was 0.01mm (SD 0.07mm), both in group A. The pooled HR per SD of annual common CIMT change (0.02 to 0.43mm) was 0.99 (95% confidence interval: 0.95–1.02) in group A, 0.98 (0.93–1.04) in group B, and 0.95 (0.89–1.04) in group C. The HR per SD of common CIMT (average of the first and the second CIMT scan, 0.09 to 0.75mm) was 1.15 (1.07–1.23) in group A, 1.13 (1.05–1.22) in group B, and 1.12 (1.05–1.20) in group C.

**Conclusions:**

We confirm that common CIMT is associated with future CVD events in individuals at high risk. CIMT change does not relate to future event risk in high-risk individuals.

## Introduction

Carotid intima media thickness (CIMT) has been debated as a screening tool[1,2] and as a surrogate marker of vascular event risk.[3] Recent publications have raised doubts about the clinical usefulness,[4] and of the surrogacy[5,6] of CIMT. In a large study on general population individuals without prevalent cardiovascular disease (CVD), we were unable to show an association between rate of change of CIMT estimated by two measurements assessed some years apart and the subsequent risk of future CVD events, although the association between CIMT, estimated as an average of the two CIMT measures at different time points, and future risk was robust and consistent.[7]

Several hypotheses have been suggested to explain this discrepancy. One credible argument is that the small CIMT change, assessed with reasonable to considerable measurement error in cohort studies, and the low event risk in the asymptomatic general population make it difficult to discern such association. Acting on this hypothesis, we aimed to study individuals at high risk, to explore whether a relation between CIMT change and CVD event risk is present. For the present analyses, we identified studies that included asymptomatic individuals with at least three CVD risk factors, asymptomatic individuals with carotid plaque, and individuals with pre-existing CVD as indicators of high risk. With individual participant data (IPD) meta-analysis we assessed the relation between CIMT, CIMT change, and subsequent vascular event risk in these groups.

## Materials and methods

To identify relevant studies for this meta-analysis, we performed a comprehensive literature research. With the search terms “intima media” AND (“myocardial infarction” OR”stroke” OR”death” OR “mortality”) we screened PubMed. In addition, we hand searched reference lists of CIMT review papers. We included publications in all languages, published until 1^st^ October 2015. Using predefined inclusion criteria (Table 1), original articles and research reports were assessed by reading both the abstracts and the full texts. When eligibility for our analysis could not be decided, we sent a short screening questionnaire to the relevant study team. If a study fulfilled all inclusion criteria, the study team was invited to join the collaboration, share their data, and participate in the project. We included cohorts with at least two CIMT scans several years apart, and a subsequent clinical follow-up.

**Table 1:** Inclusion criteria.

Population cohorts	Risk cohorts
Prospective longitudinal study design	
Investigation of a population based sample or a sample similar to the general population	Investigation of one, or including one of the following risk populations: • Individuals with at least 3 CVD risk factors • Individuals with carotid plaque • Individuals with previous MI or stroke
Well-defined and disclosed inclusion criteria and recruitment strategy	
At least two ultrasound visits where carotid IMT was determined	
A clinical follow-up after the second ultrasound visit, recording MI, stroke, death, vascular death or a subset of these.	
A minimum of 10 events per endpoint before exclusions	

The datasets underwent central plausibility checks and transformation into a standard data format with uniform variable names, units, and coding. Ordinal variables were recoded into binary balanced categories. Mean common carotid IMT (mean CCA-IMT) was calculated as the mean from all available mean CIMT measurements in the common carotid arteries, including left and right carotids, near and far wall, and all insonation angles. From the first two ultrasound visits of each study, two CIMT variables were derived: ‘average CIMT’ is the mean of the baseline and the first follow-up scan; and ‘annual CIMT change’ is the difference between the baseline and the first follow-up scan, divided by the time between scans in years. Mean CCA-IMT was used in most analyses, in some sensitivity analyses we used maximal CCA-IMT in the same way. Differences in the ultrasound measurement protocols between studies were tabulated and considered in sensitivity analyses.

We used a combined endpoint for most analyses, defined as the first event of myocardial infarction (MI), stroke (including non-traumatic intracerebral hemorrhage), or vascular death, occurring after the second ultrasound visit. For these component endpoints, the definition used in each study was adopted. When vascular death was not available in a study, total mortality was used instead. For some sensitivity analyses, we also studied the endpoints MI, stroke, and total mortality separately.

From all cohorts except one, IPD were sent to the coordinating center at Frankfurt University, where they were harmonized. The harmonized data were forwarded to the statistics center at Cambridge University for fitting of the Cox models and pooling of their estimates. One cohort (AtheroGene) was unable to forward IPD due to legal restrictions. For this cohort, the plausibility checks and the fitting of the Cox models were done locally, following the programming codes developed by the statistics center, and their estimates were sent to Cambridge for pooling.

### Statistical analyses

In order to identify individuals with high CVD risk, we used three subject groups:

A) Individuals with **three or more CVD risk factors**, including (i) male sex or age ≥ 60 years, (ii) LDL cholesterol>160mg/dl and/or lipid-lowering medication, (iii) HDL cholesterol<40mg/dl, (iv) systolic blood pressure>140mmHg, diastolic blood pressure>90mmHg and/or antihypertensive medication, (v) prediagnosed diabetes or fasting glucose>110mg/dl and/or antidiabetic medication, (vi) current smoking, (vii) triglycerides >200mg/dl and (viii) family history of CVD, **without** previous MI or stroke. This definition followed the inclusion criteria of the IMPROVE study[8];
B) Individuals with carotid plaques **without** previous MI or stroke, irrespective of the number of risk factors;
C) Individuals **with** previous MI or stroke.

From general population cohorts, individuals satisfying these respective criteria were selected. Cohorts in dedicated risk groups and hospital cohorts were included when they matched our criteria, or a relevant proportion of their individuals could be selected by our criteria.

The statistical analysis followed a pre-specified plan. For cohorts A and B, individuals who had a CVD event (MI or stroke) before the second ultrasound visit were excluded. In cohort C, individuals with endpoint events between the two ultrasound visits were excluded. For every study, considering clinical events after the second ultrasound visit, we fitted a Cox regression model for the chosen endpoint (usually combined: MI or stroke or vascular death). The hazard ratio (HR) of annual CIMT change was expressed per (within study) standard deviation (SD) of annual CIMT change. Two levels of adjustment were defined: model 1 included age, sex, and average CIMT, and model 2 included these covariates plus a large set of CVD risk factors (ethnicity, socioeconomic status, body mass index, systolic blood pressure, antihypertensive medication, total cholesterol, lipid-lowering medication, diabetes, smoking status, hemoglobin, creatinine). The log HR estimates were then pooled across all studies using random effects meta-analysis.[9] Heterogeneity between the cohorts was assessed using the I^2^ statistic.[10] If multiple studies had each less than 20 endpoint events, a Cox regression model was fitted on a merged dataset of these, stratified for the cohort, and the resulting HR was pooled with the HRs of the other cohorts. The effects of study-level variables were assessed by random effects meta-regression. All analyses were based on unimputed data (complete case analysis) since previous work had shown no material differences when using multiple imputation.[7]

The rationale and methods of the PROG-IMT project have been published beforehand.[11] The first author had full access to the data (except the IPD of AtheroGene, as explained above) and takes responsibility for their integrity. All authors have read and agreed to the manuscript as written. The PROG-IMT project and the work leading to this publication have been approved by the Ethics Committee of Frankfurt University Hospital (Geschaeftsnummer 304/13). All contributing studies had approval of their local IRB.

## Results

2513 publications were screened and 610 screening questionnaires sent. After the screening process, 60 cohorts were known to be eligible (S1 Fig). Of these, 18 declined collaboration, and 9 accepted but did not provide their dataset in time. We were able to include 23 population cohorts and 10 risk cohorts across the world. One population cohort and one risk cohort had to be excluded subsequently, because after the construction of the groups A-C, no endpoint events were left. The remaining cohorts are shown in Table 2. In group A, 23406 individuals were included, of which 3462 suffered an endpoint event. In group B, 14496 individuals with 2852 endpoint events were analyzed. Group C comprised 3628 individuals who developed 1174 endpoint events. Given our criteria, the subjects selected into group C did not overlap with those in group A or B, but A overlapped with B in 17 cohorts (by 24–79% of group A). In the Cardiovascular Health Study (CHS), individuals of Caucasian ethnicity (cohort 1) had a different follow-up regime than African Americans (cohort 2): they were considered as two separate cohorts (CHS1 and CHS2).

**Table 2:** Cohorts and subsamples.

Cohort	Cohort type	Country	Mean age (years)	Mean duration between the first 2 ultrasound visits (years)	Mean clinical follow-up after the second ultrasound visit (years)	Total number of individuals (combined endpoint events)	Number of individuals (combined endpoint events) included in A (at least 3 RF)	Number of individuals (combined endpoint events) included in B (carotid plaque)	Number of individuals (combined endpoint events) included in C (previous CVD event)
AIR[12]	Population	Sweden	58.2	3.2	5.5	391 (23)	129 (9)	106 (6)	n.a.
ARIC[13]	Population	USA	54.2	2.9	14.2	15040 (2089)	4486 (933)	3672 (707)	408 (176)
AtheroGene*[14]	Hospital	Germany	62.4	0.6	5.9	335 (36)	181 (14)	n.a.	154 (22)
BHS*[15]	Population	USA	36.3	2.5	4.5	1392 (13)^#^	179 (2)	n.a.	n.a.
Bruneck*[16]	Population	Italy	62.9	5.0	8.3	821 (113)	372 (58)	n.a.	61 (23)
CAPS[17]	Population	Germany	51.0	3.2	5.2	6972 (151)^+^	610 (40)	n.a.	95 (27)
CCCC*[18]	Population	Taiwan	54.9	5.0	6.9	3602 (116)^+^	456 (47)	250 (32)	25 (2)
CHS1[19]	Population	USA	72.8	2.9	8.5	5201 (1943)	1957 (750)	2633 (963)	777 (358)
CHS2[19]	Population	USA	73.0	6.0	5.0	687 (206)	177 (42)	217 (50)	58 (16)
CMCS[20]	Population	China	59.9	5.4	4.9	1324 (28)	369 (8)	182 (3)	43 (2)
CSN*[21]	Risk population	Italy	55.0	2.5	3.6	13843 (14)	1374 (1)	n.a.	n.a.
DIWA[22]	Population	Sweden	64.5	5.4	2.4	644 (53)	259(9)	n.a.	26 (4)
EAS[23]	Population	UK	69.0	6.6	5.3	1593 (316)	513 (29)	381 (22)	93 (11)
EPICARDIAN[24]	Population	Spain	67.7	3.1	5.6	446 (53)	156 (19)	n.a.	9 (1)
EVA[25]	Population	France	65.1	2.0	14.0	1135 (41)^#^	594 (25)	182 (13)	81 (6)
HOORN[26]	Population	Netherlands	68.2	5.2	2.7	3103 (458)	123 (1)	n.a.	7 (0)
IMPROVE[27]	Risk population	Finland, France, Italy, Netherlands, Sweden	64.2	1.2	1.8	3703(49)	2471 (41)	n.a.	n.a.
INVADE[28]	Population	Germany	67.7	2.2	3.9	3908 (602)^+^	1183 (135)	1319 (138)	408 (97)
KIHD[29]	Population	Finland	52.4	4.1	13.7	1399 (478)	669 (216)	239 (96)	98 (54)
Landecho et al.*[30]	Hospital	Spain	54.5	3.6	3.2	250 (11)	124 (5)	n.a.	n.a.
MDCS plaque substudy*[31]	Risk population	Sweden	59.5	2.1	12.2	1544 (260)	654 (157)	n.a.	31 (12)
Niguarda-Monzino*[32]	Hospital	Italy	56.2	3.4	4.1	1790 (101)	168 (7)	n.a.	n.a.
NOMAS/INVEST[33]	Population	USA	65.5	3.6	2.9	778 (27)	378 (15)	344 (18)	n.a.
OSACA-2[34]	Hospital	Japan	65.0	2.8	6.0	291 (13)	79 (2)	n.a.	109 (8)
PIVUS*[35]	Population	Sweden	70.0	5.1	1.9	1017 (114)^++^	386 (17)	398 (15)	65 (2)
PLIC[36]	Population	Italy	55.2	2.2	4.1	1782 (25)	759 (11)	343 (10)	88 (4)
RIAS[37]	Hospital	Switzerland	64.4	2.7	4.8	145 (43)	11 (4)	n.a.	54 (14)
Rotterdam[38]	Population	Netherlands	70.6	6.5	5.5	7983 (4011)^+^	1192 (317)	1227 (310)	383 (160)
SAPHIR[39]	Population	Austria	51.4	4.6	8.5	1800 (70)	445 (32)	286 (17)	39 (3)
SHIP[40]	Population	Germany	49.8	5.3	5.9	4308 (127)	1262 (71)	1006 (63)	130 (18)
SPARC*[41]	Hospital	Canada	70.3	1.1	2.1	349 (23)	182 (5)	n.a.	n.a.
Tromsø[42]	Population	Norway	59.5	6.3	8.0	4827 (850)	2091 (461)	1711 (389)	540 (176)

*included in sensitivity analyses only

^+^combined endpoint MI or stroke or death

^#^vascular death

^++^total mortality

AIR = Atherosclerosis and Insulin Resistance Study; ARIC = Atherosclerosis Risk in Communities; BHS = Bogalusa Heart Study; CAPS = Carotid Atherosclerosis Progression Study; CCCC = Chin-Shan Community Cardiovascular Cohort Study; CHS = Cardiovascular Health Study; CMCS = Chines multi-Provincial Cohort Study; CSN = The Campania Salute Network; DIWA = Diabetes and Impaired Glucose Tolerance in Women and Atherosclerosis; EAS = Edinburgh Artery Study; EVA = Étude de Vieillissement Arteriél; IMPROVE = Carotid Intima-Media Thickness and IMT-Progression as Predictors of Vascular Events in a High Risk European Population; INVADE = Interventionsprojekt zerebrovaskuläre Erkrankungen und Demenz im Landkreis Ebersberg; KIHD = Kuopio Ischemic Heart Disease Risk Factor Study; MDCS = Malmø Diet and Cancer Study; NOMAS = Northern Manhattan Study; INVEST = Oral Infections and Vascular Disease Epidemiology Study; OSACA = Osaca Follow-up Study for Atherosclerosis; PIVUS = Prospective Investigation of the Vasculature in Uppsala Seniors; PLIC = Progression of Lesions in the Intima of the Carotid; RIAS = Resistive Index in Atherosclerosis; SAPHIR = Salzburg Atherosclerosis Prevention program in subjects at High Individual Risk; SHIP = Study of Health in Pomerania; SPARC = Progression of Carotid Plaque volume predicts cardiovascular events

The distributions of average common CIMT, annual CIMT change, and of crude event rates are summarized by cohort and subgroup in S1 Table. The mean time interval between the first and second ultrasound visit was 3.57 years. Mean average common CIMT ranged from 0.68 to 1.10mm (mean 0.79mm, SD 0.16mm), and mean annual CIMT change from -0.10 to 0.05 mm/year (mean 0.01mm, SD 0.07mm, both group A). The study-specific SD for average common CIMT ranged from 0.09 to 0.75mm, the study-specific SD of annual CIMT change varied between 0.02 and 0.43mm. After the second ultrasound measurement, participants were followed up for endpoind events on average for 7.1 years. The crude event rates varied between 0.2 and 82.9 events per 1000 person years (average 19 events per 1000 person years).

**Fig 1:**
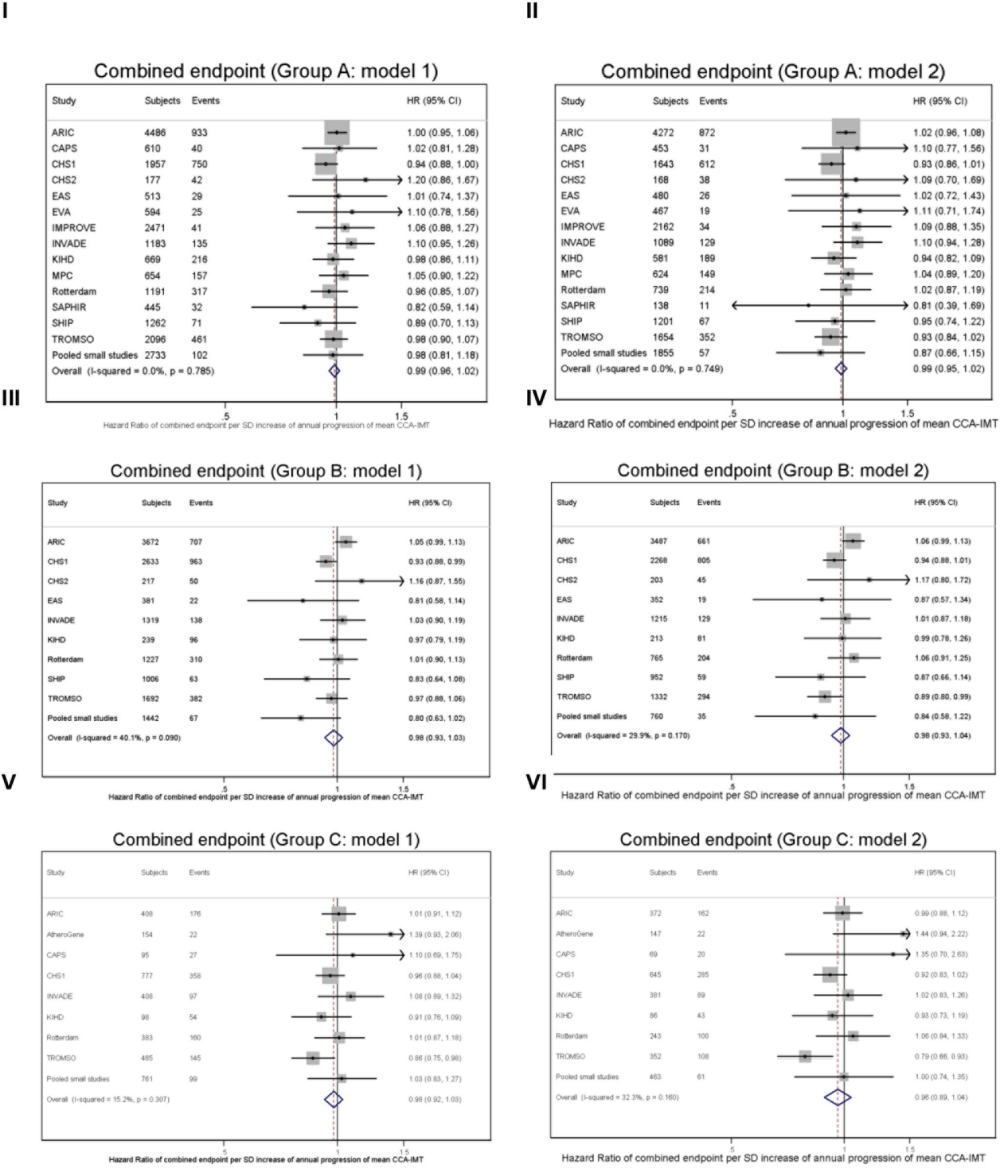
Forest plots of the HR of the combined endpoint per one SD of annual mean CCA-IMT change (with 95% CIs). Panel I: Group A (asymptomatic individuals with three or more CVD risk factors), HR adjusted for age, sex and average mean CCA-IMT (model 1). Panel II: Group A (asymptomatic individuals with three or more CVD risk factors), HR adjusted for age, sex, average mean CCA-IMT and other CVD risk factors (model 2). Panel III: Group B (asymptomatic individuals with carotid plaques), HR adjusted for age, sex and average mean CCA-IMT (model 1). Panel IV: Group B (asymptomatic individuals with carotid plaques), HR adjusted for age, sex, average mean CCA-IMT and other CVD risk factors (model 2). Panel V: Group C (individuals with previous CVD events), HR adjusted for age, sex and average mean CCA-IMT (model 1). Panel VI: Group C (individuals with previous CVD events), HR adjusted for age, sex, average mean CCA-IMT and other CVD risk factors (model 2).

**Fig 2:**
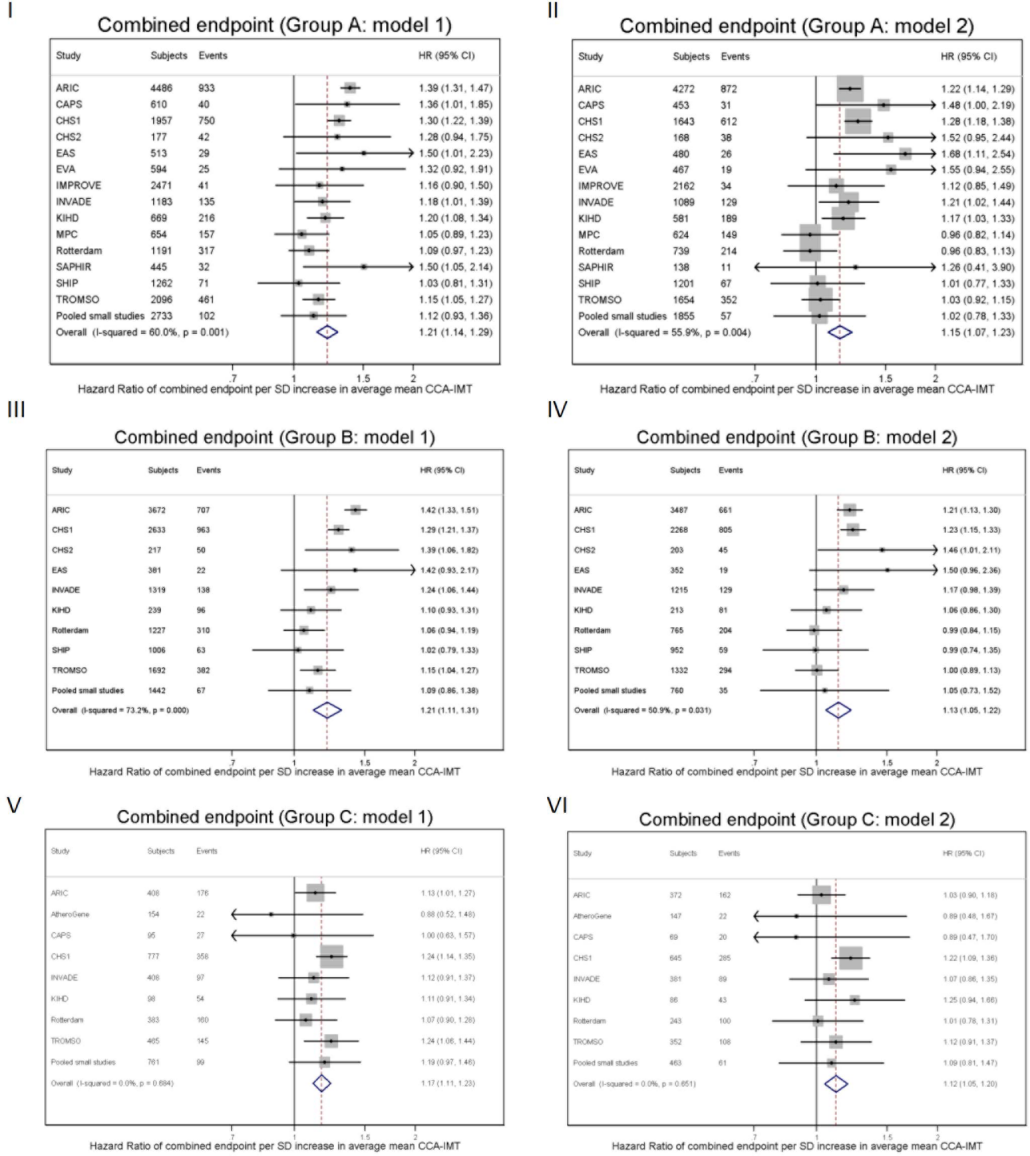
Forest plots of the HR of the combined endpoint per one SD of average mean CCA-IMT (with 95% CIs). Panel I: Group A (asymptomatic individuals with three or more CVD risk factors), HR adjusted for age, sex and annual mean CCA-IMT change (model 1). Panel II: Group A (asymptomatic individuals with three or more CVD risk factors), HR adjusted for age, sex, annual mean CCA-IMT change and other CVD risk factors (model 2). Panel III: Group B (asymptomatic individuals with carotid plaques), HR adjusted for age, sex and annual mean CCA-IMT change (model 1). Panel IV: Group B (asymptomatic individuals with carotid plaques), HR adjusted for age, sex, annual mean CCA-IMT change and other CVD risk factors (model 2). Panel V: Group C (individuals with previous CVD events), HR adjusted for age, sex and annual mean CCA-IMT change (model 1). Panel VI: Group C (individuals with previous CVD events), HR adjusted for age, sex, annual mean CCA-IMT change and other CVD risk factors (model 2).

In Fig 1 we show the association between annual common CIMT change and the combined endpoint in all three groups. There was no significant relation in any group, whether adjusted for CVD risk factors or not. Between the cohorts, I^2^ statistics indicated no substantial heterogeneity. Fig 2 displays the relation between average common CIMT and the combined endpoint. In all three groups, there were significant and consistent positive associations, which attenuated on adjustment for CVD risk factors; the HRs were somewhat heterogeneous between the cohorts (statistically significant in groups A and B). Sensitivity analyses showed very similar results for the separate endpoints MI, stroke, and total mortality; and also for maximal CCA-IMT (shown for group A in S2–S5 Figs). To allow for a non-linear association, we assessed the association between CIMT and risk in Cox regression model including a quadratic term of CIMT change. We found a HR of 0.98 (95% CI 0.95–1.02) per SD of annual mean CCA-IMT progression (I^2^ = 10.9%, p for heterogeneity = 0.331) and of 1.22 (1.14–1.30) per SD of average mean CCA-IMT (i^2^ = 60.6%, p for heterogeneity = 0.001) for the combined endpoint.

In three cohorts (ARIC, INVADE, KIHD), CIMT measurements were available from four visits. In these cohorts, we estimated the correlation between the annual common CIMT change from visit 1 to visit 2, with the annual CIMT change from visit 3 to visit 4. This correlation was -0.021 in ARIC (p = 0.60), -0.065 in INVADE (p = 0.11), and -0.082 in KIHD (p = 0.11).

**Fig 3:**
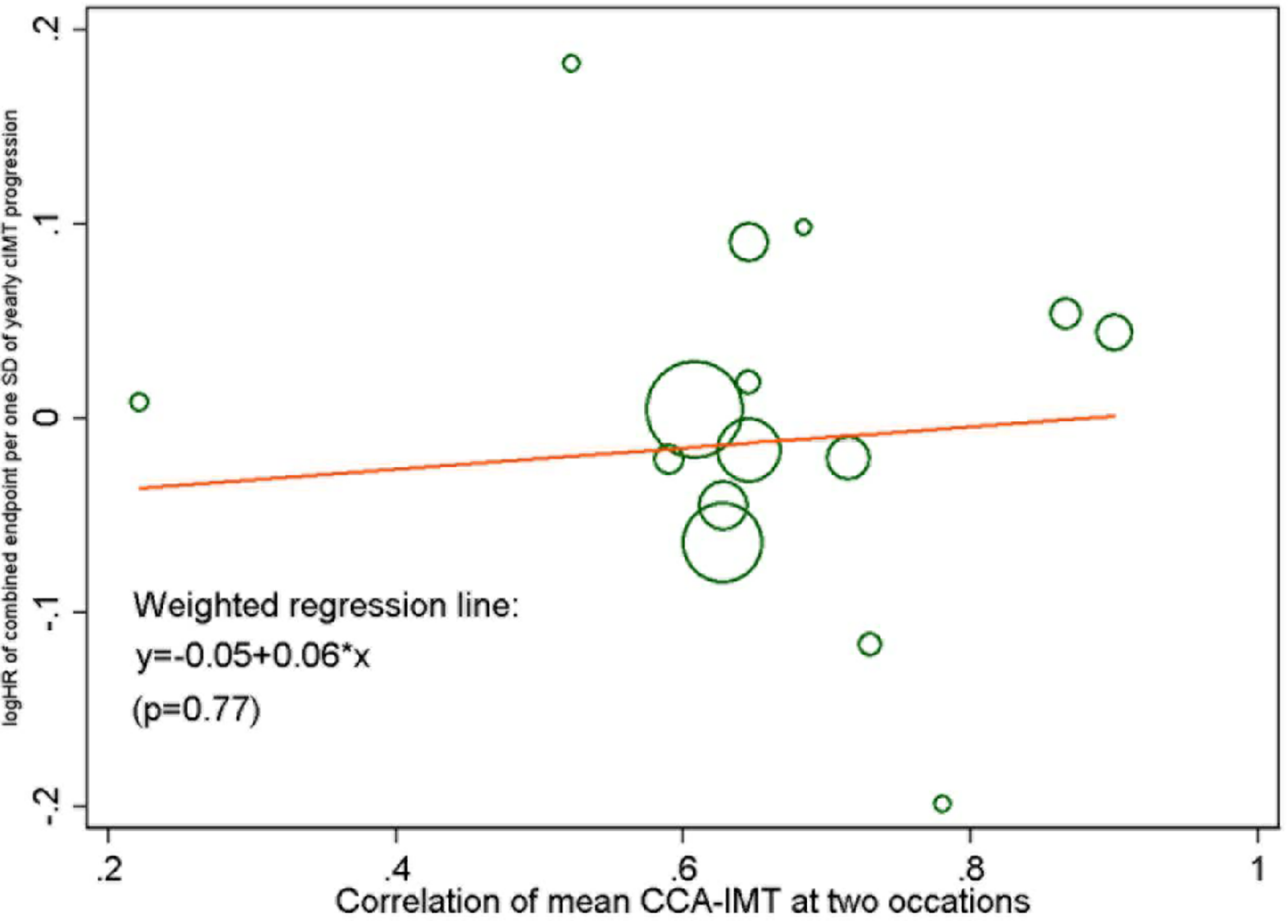
Meta-regression plot for the HR (combined endpoint) per SD of annual mean CCA-IMT change (model 1), by the correlation of baseline and follow-up common CIMT. The size of each circle represents the precision of the log HR.

We studied the influence of the accuracy of CIMT measurement on the association between annual common CIMT change and risk in meta-regression analyses. There was no significant relation between the year of the study start and the HR for the combined endpoint per SD of annual common CIMT change (S6 Fig). Fig 3 shows the meta-regression of the correlation between the two CIMT measurements (as an indicator of measurement precision) and the HR, again with no significant relation. To assess the influence of the ultrasound protocol, we repeated the meta-analysis for individuals with prevalent carotid plaques (group B) and grouped the cohorts into those where CIMT measurement included carotid plaques and those where plaques were avoided (S7 Fig). The pooled HR for the combined endpoint did not differ between these two groups.

## Discussion

Within a global collaborative project (see www.prog-imt.org), we managed to amass a large proportion of the worldwide available data in high-risk individuals (52% of all eligible cohorts), in order to assess the association between common CIMT change, and vascular event risk. Even in the selected high-risk individuals studied here, we were unable to demonstrate any association. In contrast, the known association between CIMT and vascular event risk was reproduced in a very consistent way.

There may be both methodological reasons and biological explanations for this discrepancy. One key methodological finding is that, even in high-risk populations, annual CIMT change was not a stable property of individuals, and therefore not a reproducible biomarker. When we compared–in three cohorts with the necessary data–CIMT change from visit 1 to visit 2 with CIMT change from visit 3 to visit 4 (all several years apart), we found no correlation.

But what is behind this lack of reproducibility? As can be seen in S1 Table, the range of common CIMT change, compared to CIMT, is very wide both within and between cohorts, indicating that measurement error is a major issue. For example in group A, average common CIMT is 3 to 8fold higher than its study-specific standard deviation, whereas annual common CIMT change is always smaller than its SD. It is plausible that the small systematic changes of CIMT within a few years are dwarfed by measurement error and random fluctuations.

A key problem of measuring CIMT change is to pinpoint the exact same measurement site in the carotid artery, years after the first measurement, and often done by a different technician. Despite multiple provisions in the ultrasound protocols, this seems to be an unresolved problem. As many of the studies shown here–and in particular the largest of them–were planned and started decades ago, we may hope that the newest studies and trials perform better. At least among the available cohorts, neither the year of study start, nor the accuracy of CIMT measurement had any significant effect on the CIMT-risk association we studied.

A plausible biological reason for these null findings is the complexity of the atherosclerotic process. CIMT reflects not only atherosclerosis, but also an adaptive component of the muscular wall, sometimes referred to as ‘remodelling’ [43–48]. In addition, in patients with high event risk, focal plaques may superimpose CIMT. Although overall, CIMT and plaques are progressing in parallel, there are individuals with low CIMT and impressive plaques (focal type), and vice versa (diffuse type of atherosclerosis).[49] Risk factors can act differently on CIMT and plaques,[50–52] and the association between plaque and CVD event risk may be closer than between CIMT and risk.[53]

In sensitivity analyses we studied cohorts where plaques were excluded from the CIMT measurement separately, but found no significant differences. However, it may not always be possible to avoid focal lesions when they are very distinct, and in the ultrasound measurement, the differentiation between diffuse (CIMT) and focal (plaque) atherosclerotic lesions is not clear-cut. So perhaps an isolated investigation of CIMT is too limited. Unfortunately, given the complex spatial structure of plaques, it is much more difficult to study plaque and plaque change, compared to CIMT. The standardization process for plaque measurement is years behind CIMT, where there is at least an international consensus.[54] Moreover, the amount of data that is available to analyze plaque change with standardized measurements is considerably lower than for CIMT change.

Linked with the previous argument, individuals with multiple risk factors, with carotid plaques, and stroke or MI patients, are often subjected to intensive risk factor management, life style modifications, and polypharmacy. Although we attempted to adjust for antihypertensive and lipid lowering medication, complex interactions between risk factors, nutrition, exercise, drugs and CIMT may obscure the association between CIMT and risk.

It is very important to distinguish between the ‘surrogacy’ at an individual level, as assessed here, and surrogacy at a group level, which is important for the interpretation of clinical trials about CIMT change. In this paper we addressed whether individuals whose CIMT progresses have higher subsequent event risk. For the interpretation of clinical trials with the endpoint CIMT change, we need to know whether a group of individuals treated with a drug whose CIMT progressed on average less than another group treated with another drug (or placebo), exhibits a lower event risk in the same period. This latter question has not been answered satisfactorily yet, as the current findings are contradictory.[5,6] The criteria of surrogacy in clinical trials, that is whether the effects of interventions on CIMT parallel the effects on risk, will be addressed in stage 3 of the PROG-IMT project.[11]

### Limitations

It may be argued that many of the individuals included here were already studied in our previous work on general population cohorts.[7] Three arguments counteract this point: First, we selected only individuals at high cardiovascular risk out of these population cohorts. This could well have improved the ratio between the hypothesized association, and measurement error. Second, we added a number of population based studies [18, 20, 22, 24, 26, 39], risk cohorts [21, 27, 31] and hospital cohorts [14, 30, 32, 34, 37, 41] since the above cited publication. These new cohorts (15 of 31) comprise 33% of the sample size, and 10% of the endpoint events. Third, group C included only individuals that were explicitly excluded from the analyses of our previous work.

## Conclusions

Although common CIMT is associated with future CVD event risk, this is not apparently true for common CIMT change over time. Reasons may include the complexity of atherosclerotic process, and technical limits of current CIMT measurement.

Do these null findings mean that CIMT (change) is not scientifically useful? Our results confirm that CIMT is still a very useful biomarker, with close associations with both risk factors and future endpoints. The change of CIMT, however, should be interpreted with care.

## Supporting information

S1 Table Distribution of average mean CCA-IMT, annual change of mean CCA-IMT, and crude event rates by cohort and subgroup. *mean CCA-IMT not available, maximal CCA-IMT used instead.
^&^combined endpoint not available, total mortality used instead.
(DOCX)

S2 Table Study-specific details of the ultrasound protocols. ^+^plaques purposely included.
^#^internal landmarks in computer aided navigation aid.
^++^2D images extracted from 3D dataset.
n.s. = not specified.
(DOCX)

S3 Table Data sharing restrictions, availability and contacts by study. (PDF)

S4 Table List of collaborators within the PROG-IMT study group, current from 10th June 2016. (PDF)

S1 Fig Flowchart on available studies. (DOCX)

S2 Fig Forest plots of the HR of MI in group A (asymptomatic individuals with three or more CVD risk factors) with 95% Cls. Left panel: HR for MI per one SD of annual mean CCA-IMT change, adjusted for age, sex and average mean CCA-IMT (model 1).
Right panel: HR for MI per one SD of average mean CCA-IMT, adjusted for age, sex and annual mean CCA-IMT change (model 1).
(DOCX)

S3 Fig Forest plots of the HR of stroke in group A (asymptomatic individuals with three or more CVD risk factors) with 95% Cls. Left panel: HR for stroke per one SD of annual mean CCA-IMT change, adjusted for age, sex and average mean CCA-IMT (model 1).
Right panel: HR for stroke per one SD of average mean CCA-IMT, adjusted for age, sex and annual mean CCA-IMT change (model 1).
(DOCX)

S4 Fig Forest plots of the HR of total mortality in group A (asymptomatic individuals with three or more CVD risk factors) with 95% Cls. Left panel: HR for total mortality per one SD of annual mean CCA-IMT change, adjusted for age, sex and average mean CCA-IMT (model 1).
Right panel: HR for total mortality per one SD of average mean CCA-IMT, adjusted for age, sex and annual mean CCA-IMT change (model 1).
(DOCX)

S5 Fig Forest plots of the HR of the combined endpoint in group A (asymptomatic individuals with three or more CVD risk factors) for maximal CCA-IMT with 95% Cls. Left panel: HR for the combined endpoint per one SD of annual maximal CCA-IMT change, adjusted for age, sex and average maximal CCA-IMT (model 1).
Right panel: HR for the combined endpoint per one SD of average maximal CCA-IMT, adjusted for age, sex and annual maximal CCA-IMT change (model 1).
(DOCX)

S6 Fig Meta-regression plots for the HR (combined endpoint) per SD of annual mean CCA-IMT change, by the year of the study start for group A cohorts. The size of each circle represents the precision of the log HR.
Left panel: Model 1 (HR adjusted for age, sex, and average mean CCA-IMT): weighted regression line y = 7.07+0.004*x (p = 0.34).
Right panel: Model 2 (HR adjusted for age, sex, average mean CCA-IMT and other CVD risk factors): weighted regression line y = -9.43+0.005*x (p = 0.32).
(DOCX)

S7 Fig Forest plots of the HR of the combined endpoint per one SD of annual mean CCA-IMT change, grouped by IMT measurement protocol, with 95% Cls. Group B (asymptomatic individuals with carotid plaques), HR adjusted for age, sex and average mean CCA-IMT (model 1).
(DOCX)
