# Correction: Predictive value for cardiovascular events of common carotid intima media thickness and its rate of change in individuals at high cardiovascular risk - Results from the PROG-IMT collaboration

**DOI:** 10.1371/journal.pone.0204633

**Published:** 2018-09-20

**Authors:** Matthias W. Lorenz, Lu Gao, Kathrin Ziegelbauer, Giuseppe Danilo Norata, Jean Philippe Empana, Irene Schmidtmann, Hung-Ju Lin, Stela McLachlan, Lena Bokemark, Kimmo Ronkainen, Mauro Amato, Ulf Schminke, Sathanur R. Srinivasan, Lars Lind, Shuhei Okazaki, Coen D. A. Stehouwer, Peter Willeit, Joseph F. Polak, Helmuth Steinmetz, Dirk Sander, Holger Poppert, Moise Desvarieux, M. Arfan Ikram, Stein Harald Johnsen, Daniel Staub, Cesare R. Sirtori, Bernhard Iglseder, Oscar Beloqui, Gunnar Engström, Alfonso Friera, Francesco Rozza, Wuxiang Xie, Grace Parraga, Liliana Grigore, Matthieu Plichart, Stefan Blankenberg, Ta-Chen Su, Caroline Schmidt, Tomi-Pekka Tuomainen, Fabrizio Veglia, Henry Völzke, Giel Nijpels, Johann Willeit, Ralph L. Sacco, Oscar H. Franco, Heiko Uthoff, Bo Hedblad, Carmen Suarez, Raffaele Izzo, Dong Zhao, Thapat Wannarong, Alberico Catapano, Pierre Ducimetiere, Christine Espinola-Klein, Kuo-Liong Chien, Jackie F. Price, Göran Bergström, Jussi Kauhanen, Elena Tremoli, Marcus Dörr, Gerald Berenson, Kazuo Kitagawa, Jacqueline M. Dekker, Stefan Kiechl, Matthias Sitzer, Horst Bickel, Tatjana Rundek, Albert Hofman, Ellisiv B. Mathiesen, Samuela Castelnuovo, Manuel F. Landecho, Maria Rosvall, Rafael Gabriel, Nicola de Luca, Jing Liu, Damiano Baldassarre, Maryam Kavousi, Eric de Groot, Michiel L. Bots, David N. Yanez, Simon G. Thompson, on behalf of the PROG-IMT study group

An affiliation for Moise Desvarieux is missing. In addition to affiliation #22, Moise Desvarieux is affiliated with: METHODS Core, Centre de Recherche Epidémiologie et Statistique Paris Sorbonne Cité (CRESS), Institut National de la Santé et de la Recherche Médicale (INSERM) UMR 1153, Paris France.
