# Supplementary material for: Predictive value for cardiovascular events of common carotid intima media thickness and its rate of change in individuals at high cardiovascular risk – Results from the PROG-IMT collaboration
Source: PLoS One. 2018 Apr 12;13(4):e0191172. doi: 10.1371/journal.pone.0191172 (PMC5896895; doi:10.1371/journal.pone.0191172)
Supplement: S1 Table — *mean CCA-IMT not available, maximal CCA-IMT used instead. &combined endpoint not available, total mortality used instead. (DOCX) [file pone.0191172.s001.docx]

S1 Table: Distribution of average mean CCA-IMT, annual change of mean CCA-IMT, and crude event rates by cohort and subgroup.

| **Cohort** | **Group A (at least 3 RF)** | | | | **Group B (carotid plaque)** | | | | **Group C (previous CVD event)** | | | |
| --- | --- | --- | --- | --- | --- | --- | --- | --- | --- | --- | --- | --- |
|  | **Average of**  **mean CCA- IMT**  **mean (SD)** | **Correlation of 1^st^ and 2^nd^ mean CCA-IMTvalue (r)** | **Annual change of**  **mean CCA-IMT**  **mean (SD)** | **Combined endpoint events per**  **1000 person**  **Years (95% CI)** | **Average of**  **mean CCA- IMT**  **mean (SD)** | **Correlation of 1^st^ and 2^nd^ mean CCA-IMTvalue (r)** | **Annual change of**  **mean CCA-IMT**  **mean (SD)** | **Combined endpoint events per**  **1000 person**  **Years (95% CI)** | **Average of**  **mean CCA- IMT**  **mean (SD)** | **Correlation of 1^st^ and 2^nd^ mean CCA-IMTvalue (r)** | **Annual change of**  **mean CCA-IMT**  **mean (SD)** | **Combined endpoint events per**  **1000 person**  **Years (95% CI)** |
| **AIR** | 0.81 (0.13) | 0.75 | 0.00 (0.03) | 12.88 (6.70, 24.76) | 0.82 (0.11) | 0.68 | 0.00 (0.03) | 10.26 (4.61, 22.85) | n.a |  |  |  |
| **ARIC** | 0.70 (0.12) | 0.61 | 0.01 (0.04) | 15.41 (14.45, 16.43) | 0.71 (0.13) | 0.65 | 0.01 (0.04) | 14.13 (13.12,15.21) | 0.73 (0.15) | 0.56 | 0.01 (0.05) | 38.85 (33.51, 45.03) |
| **AtheroGene** | 0.76 (0.17) | 0.50 | 0.20 (0.40) | 12.54 (13.77, 31.98) | n.a |  |  |  | 0.77 (0.22) | 0.70 | 0.20 (0.33) | 23.54 (17.31, 39.49) |
| **BHS** | 0.81 (0.14)* | 0.65 | 0.00 (0.06)* | 2.54 (0.64, 10.17) | n.a |  |  |  | n.a |  |  |  |
| **Bruneck** | 0.99 (0.18)* | 0.76 | 0.03 (0.03)* | 18.50 (14.30, 23.93) | n.a |  |  |  | 1.10 (0.17)* | 0.70 | 0.02 (0.03)* | 57.09 (37.94, 85.92) |
| **CAPS** | 0.79 (0.14) | 0.65 | 0.00 (0.04) | 12.78 (9.37, 17.42) | n.a |  |  |  | 0.89 (0.32) | 0.64 | 0.01 (0.11) | 63.34 (43.44, 92.36) |
| **CCCC** | 0.80 (0.20)* | 0.43 | 0.02 (0.06)* | 14.27 (10.72, 18.99) | 0.90 (0.22) | 0.37 | 0.04 (0.06) | 17.00 (11.95, 24.17) | 0.92 (0.26)* | 0.62 | 0.03 (0.06)* | 92.77 (54.95, 156.65) |
| **CHS1** | 0.89 (0.15) | 0.63 | 0.01 (0.05) | 44.81 (41.71, 48.13) | 0.89 0.15) | 0.63 | 0.00 (0.05) | 42.37 (39.78, 45.14) | 0.92 (0.18) | 0.62 | 0.00 (0.06) | 64.09 (57.78, 71.08) |
| **CHS2** | 0.94 (0.16) | 0.52 | 0.01 (0.03) | 45.46 (33.60, 61.51) | 0.94 (0.16) | 0.54 | 0.01 (0.03) | 45.23(34.27, 59.67) | 0.94 (0.18) | 0.49 | 0.02 (0.04) | 55.61 (34.06, 90.77) |
| **CMCS** | 0.87 (0.24) | 0.53 | 0.04 (0.05) | 4.48 (2.24, 8.96) | 0.92 (0.27) | 0.59 | 0.05 (0.05) | 3.37 (1.08, 10.45) | 0.90 (0.27) | 0.47 | 0.06 (0.07) | 9.43 (2.36, 37.72) |
| **CSN** | 1.10 (0.34)* | 0.84 | 0.03 (0.43) | 0.22 (0.03, 1.55) | n.a |  |  |  | n.a |  |  |  |
| **DIWA** | 0.89 (0.15) | 0.76 | 0.00 (0.02) | 14.68(7.64, 28.21) | n.a |  |  |  | 0.91 (0.15) | 0.61 | 0.00 (0.03) | 67.33 (25.27,179.40) |
| **EAS** | 0.86 (0.18) | 0.22 | 0.03 (0.05) | 10.01 (6.95, 14.40) | 0.86 (0.16) | 0.31 | 0.03 (0.04) | 10.43 (6.87, 15.85) | 0.89 (0.23) | 0.37 | 0.03 (0.05) | 23.33 (12.92, 42.12) |
| **EPICARDIAN** | 0.79 (0.21) | 0.08 | -0.10 (0.13) | 20.98 (13.38, 32.90) | n.a |  |  |  | 0.77 (0.23) | 0.43 | -0.13 (0.11) | 47.96 (15.47, 138.70) |
| **EVA** | 0.68 (0.11) | 0.68 | 0.01 (0.05) | 2.99 (2.02, 4.42) | 0.71 (0.12) | 0.70 | 0.01 (0.05) | 5.31 (3.08, 9.15) | 0.68 (0.11) | 0.57 | 0.01 (0.06) | 5.30 (2.38, 11.79) |
| **HOORN** | 0.86 (0.12) | 0.30 | 0.01 (0.04) | 2.99 (0.42, 21.24) | n.a |  |  |  | 0.94 (0.15) | -0.05 | -0.01 (0.06) | 0 |
| **IMPROVE** | 0.76 (0.15) | 0.87 | 0.01 (0.06) | 8.95 (6.60,12.15) | n.a |  |  |  | n.a |  |  |  |
| **INVADE** | 0.85 (0.18) | 0.65 | 0.01 (0.08) | 28.85 (24.37, 34.15) | 0.83 (0.17) | 0.62 | 0.01 (0.08) | 26.2 (22.2, 31.0) | 0.89 (0.19) | 0.61 | 0.01 (0.09) | 62.26 (51.02, 75.97) |
| **KIHD** | 0.82 (0.16) | 0.72 | 0.03 (0.04) | 23.26 (20.36, 26.58) | 0.90 (0.19) | 0.72 | 0.03 (0.04) | 32.67 (26.75, 39.91) | 0.91 (0.22) | 0.62 | 0.03 (0.05) | 58.31 (44.66, 76.14) |
| **Landecho et al.** | 0.71 (0.14) | 0.57 | 0.00 (0.06) | 13.05 (5.43, 31.34) | n.a |  |  |  | n.a |  |  |  |
| **MDCS plaque substudy (MPC)** | 0.90 (0.18) | 0.70 | 0.05( 0.09) | 20.69 (17.69, 24.19) | n.a |  |  |  | 0.90 (0.22) | 0.79 | 0.02 (0.08) | 41.31 (23.46, 72.75) |
| **Niguarda-Monzino** | 0.99 (0.32) | 0.84 | 0.03 (0.14) | 9.53 (4.53, 19.99) | n.a |  |  |  | n.a |  |  |  |
| **NOMAS/INVEST** | 0.72 (0.08) | 0.42 | 0.01 (0.03) | 13.64 (8.22, 22.62) | 0.75 (0.08) | 0.36 | 0.01 (0.03) | 18.06 (11.38, 28.67) | n.a |  |  |  |
| **OSACA-2** | 0.88 (0.20) | 0.45 | 0.02 (0.11) | 4.28 (1.07, 17.12) | n.a |  |  |  | 0.87 (0.19) | 0.50 | 0.03 (0.10) | 12.13 (6.06, 24.25) |
| **PIVUS** | 0.94 (0.15) | 0.56 | 0.01 (0.03) | 23.89 (14.85, 38.43) | 0.95 (0.15) | 0.58 | 0.01 (0.03) | 20.26 (12.21, 33.61) | 0.92 (0.16) | 0.34 | 0.00 (0.04) | 16.16 (4.04, 64.61) |
| **PLIC** | 0.70 (0.14) | 0.84 | 0.01 (0.04) | 3.53 (1.96, 6.38) | 0.73 (0.14) | 0.81 | 0.01 (0.04) | 6.98 (3.76, 12.98) | 0.72 (0.14) | 0.73 | 0.02 (0.05) | 11.24 (4.22, 29.94) |
| **RIAS** | 0.80 (0.13) | 0.95 | 0.05 (0.09) | 66.35 (24.90, 176.80) | n.a |  |  |  | 0.85 (0.14) | 0.83 | 0.02 (0.09) | 46.20 (27.35, 78.00) |
| **Rotterdam** | 0.83 (0.15) | 0.63 | 0.01 (0.02) | 48.57 (43.50, 54.22) | 0.84 (0.15) | 0.60 | 0.01 (0.02) | 46.58 (41.67, 52.06) | 0.85 (0.16) | 0.64 | 0.01 (0.02) | 83.2 (71.22, 97.01) |
| **SAPHIR** | 0.83 (0.13) | 0.77 | 0.02 (0.02) | 8.45 (5.97, 11.96)^&0^ | 0.88 (0.14) | 0.75 | 0.02 (0.02) | 6.96 (4.33, 11.20) | 0.86 (0.15) | 0.76 | 0.01 (0.03) | 8.84 (2.85, ,27.39) |
| **SHIP** | 0.80 (0.15) | 0.73 | 0.01 (0.02) | 9.50 (7.53, 11.99) | 0.82 (0.15) | 0.72 | 0.01 (0.02) | 10.74 (8.38, 13.74) | 0.86 (0.17) | 0.78 | 0.01 (0.03) | 24.91(15.69, 39.54) |
| **SPARC** | 0.94 (0.16) | 0.70 | 0.00 (0.13) | 13.45 (5.59, 32.30) | n.a |  |  |  | n.a |  |  |  |
| **Tromsø** | 0.82 (0.14) | 0.65 | 0.01 (0.02) | 28.60 (26.11, 31.33) | 0.84 (0.15) | 0.63 | 0.00 (0.02) | 29.41 (26.61, 32.52) | 0.85 (0.15) | 0.60 | 0.00 (0.02) | 42.87 (36.44, 50.45) |

*mean CCA-IMT not available, maximal CCA-IMT used instead

^&^combined endpoint not available, total mortality used instead
