# Supplementary material for: Predictive value for cardiovascular events of common carotid intima media thickness and its rate of change in individuals at high cardiovascular risk – Results from the PROG-IMT collaboration
Source: PLoS One. 2018 Apr 12;13(4):e0191172. doi: 10.1371/journal.pone.0191172 (PMC5896895; doi:10.1371/journal.pone.0191172)
Supplement: S2 Table — +plaques purposely included. #internal landmarks in computer aided navigation aid. ++2D images extracted from 3D dataset. n.s. = not specified. (DOCX) [file pone.0191172.s002.docx]

S2 Table: Study-specific details of the ultrasound protocols

| **Cohort** | **CCA landmark** | **CCA length** | **Location of meaurements** | **Avoid**  **plaques** | **ECG**  **gated** | **Angle**  **control** | **Multiple**  **scans** | **Central**  **reading** | **Edge detection algorithm** |
| --- | --- | --- | --- | --- | --- | --- | --- | --- | --- |
| AIR | Beginning of bulbar widening | 1cm | Right&left far wall | No | Yes | No | No | Yes | Yes |
| ARIC | Beginning of bulbar widening | 1cm | Right&left near and far wall | No | Yes | Yes | Yes | Yes | No |
| AtheroGene | Beginning of bulbar widening |  | Right&left far wall | No | No | No | No | No | No |
| BHS | Beginning of bulbar widening | 1cm | Right&left far wall | No | Yes | Yes | Yes | Yes | No |
| Bruneck | Beginning of bulbar widening | 3cm | Right&left near and far wall | Yes | Yes | Yes | No | No | No |
| CAPS | 2cm proximal to the tip of the flow divider | up to 4cm | Right&left far wall | Not specified | No | No | No | Yes | Yes |
| CCCC | 1cm proximal to the tip of the flow divider | 1cm | Right&left far wall | Yes | Yes | Yes | Yes | No | No |
| CHS | Beginning of bulbar widening or (if not determinable) 1cm proximal to the tip of the flow divider | 1cm | Right&left near and far wall | No | No | No | No | Yes | No |
| CMCS | Beginning of bulbar widening | Three single measurements each in 3 segments of 1cm | Right&left near and far wall | Yes | No | No | No | Yes | No |
| CSN | Beginning of bulbar widening |  | Right&left near and far wall | n.s. | No | No | No | Yes | No |
| DIWA | Beginning of bulbar widening | 1cm | Right&left far wall | No^+^ | Yes | No | No | Yes | Yes |
| EAS | Beginning of bulbar widening | 0,5cm | Right&left far wall | Yes | No | No | No | Yes | No |
| EPICARDIAN | Beginning of bulbar widening | 1cm | Right&left near and far wall | Yes | No | Yes | Yes | No | No |
| EVA | 2cm proximal to the tip of the flow divider | 2cm | Right&left far wall | Yes | No | Yes | No | Yes | Yes |
| Hoorn | Beginning of bulbar widening | 1cm | Right far wall | Yes | Yes | Yes | Yes | Yes | Yes |
| IMPROVE | Beginning of bulbar widening | 1cm | Right&left far wall | No | No | No | Yes | No | No |
| INVADE | 1cm proximal to the tip of the flow divider | 1cm | Right&left far wall | Yes | No | Yes | Yes | Yes | Yes |
| KIHD | Beginning of bulbar widening | 1-5cm | Right&left far wall | No | No | No | No | Yes | Yes |
| Landecho et al. | Beginning of bulbar widening | 1cm | Right&left near and far wall | Yes | No | No | No | No | No |
| MDCS plaque substudy (MPC) | Beginning of bulbar widening | 1cm | Right near and far wall | n.s. | Yes | No | No | Yes | Yes |
| Niguarda-Monzino | Beginning of bulbar widening | 1cm | Right&left near and far wall | No | No | No | Yes | No | No |
| NOMAS/INVEST | 1cm proximal to the tip of the flow divider | 1cm | Right&left near and far wall | Yes | No | Yes^#^ | No | Yes | Yes |
| OSACA-2 | No landmark described | At the site of greatest IMT, and 1cm up- and downstream | Right&left near and far wall | No* | No | No | No | Yes | No |
| PIVUS | Beginning of bulbar widening | 1cm | Right&left far wall | n.s. | Yes | No | No | Yes | Yes |
| PLIC | 2cm proximal to the tip of the flow divider | 2cm | Right&left far wall | Yes | Yes | No | No | Yes | Yes |
| RIAS | Beginning of bulbar widening | 1cm | Right&left near and far wall | Yes | Yes | No | Yes | No | No |
| Rotterdam | Beginning of bulbar widening | 1cm | Right&left near and far wall | No | Yes | No | Yes | Yes | Yes |
| SAPHIR | 8 mm proximal to the tip  of the flow divider | 8mm | Right&left near and far wall | Yes | No | Yes | Yes | No | No |
| SHIP | Beginning of bulbar widening | 1cm | Right&left far wall | No | No | No | No | Yes | Yes |
| SPARC^++^ | Beginning of bulbar widening | 1cm | Far wall bilateral | Yes | No | No | No | Yes | No |
| Tromsø | Beginning of bulbar widening | 1cm | Right near and far wall | No^+^ | Yes | No | Yes | Yes | Yes |

^+^plaques purposely included

^#^internal landmarks in computer aided navigation aid

^++^2D images extracted from 3D dataset

n.s.=not specified
