# Supplementary material for: Predictive value for cardiovascular events of common carotid intima media thickness and its rate of change in individuals at high cardiovascular risk – Results from the PROG-IMT collaboration
Source: PLoS One. 2018 Apr 12;13(4):e0191172. doi: 10.1371/journal.pone.0191172 (PMC5896895; doi:10.1371/journal.pone.0191172)
Supplement: S3 Table — (PDF) [file pone.0191172.s003.pdf]

Study	Third party owned	Restrictions against public data sharing	Availability and contact
AIR	No	Swedish law regarding personal information, PROG-IMT policy	Data can be shared on a scientific collaborative basis (Contact: Goran.Bergstrom@hjl.gu.se)
ARIC	Yes	None, available via BioLincc	https://biolincc.nhlbi.nih.gov/studies/aric/
AtheroGene	No	The ethical vote does not cover data tranfer outside the study center	Transfer of data is strictly prohibited. For the purpose of the current analysis, the AtheroGene data were analysed by a member of the Mainz biometric team – Irene Schmidtmann – following specific statistc models provided by central statistics of PROG-IMT*. Requests on similar collaboration may be considered (Contact: s.blankenberg@uke.de)
BHS	Yes	PROG-IMT policy	Data can be shared on a scientific collaborative basis (Contact: berenson@tulane.edu)
Bruneck	No	Open access to the study data is not covered by the signed informed agreements of the study participants and the approval of the ethics committee.	Anonymised data are available on request for researchers who meet the criteria for access to confidential data, including accreditation as approved researcher by the local data sharing committee and signing of a data sharing agreement with the study. (Contact: Vascage@i-med.ac.at)
CAPS	No	PROG-IMT policy	Data can be shared on a scientific collaborative basis (Contact: matthias.lorenz@em.uni-frankfurt.de)
CCCC	No	PROG-IMT policy	Data can be shared on a scientific collaborative basis (Contact: klchien@ntu.edu.tw)
CHS	Yes	None, available via BioLincc	https://biolincc.nhlbi.nih.gov/studies/chs/
CMCS	No	PROG-IMT policy	Data can be shared on a scientific collaborative basis (Contact: ejingliu@163.com)
CSN	No	PROG-IMT policy	Data can be shared on a scientific collaborative basis (requests to the CSN steering committee, contact: rafizzo@unina.it)
DIWA	No	Swedish law regarding personal information, PROG-IMT policy	Data can be shared on a scientific collaborative basis (Contact: Goran.Bergstrom@hjl.gu.se)
EAS	No	The study has to approve of such sharing for ethical reason, PROG-IMT policy	Data can be shared on a scientific collaborative basis (ethical committee of Edinburgh University, contact: Stela.McLachlan@ed.ac.uk)
**Study**	**Third party**	**Restrictions against public data sharing**	**Availability and contact**

	owned		
EPICARDIAN	No	PROG-IMT policy	Data can be shared on a scientific collaborative basis (Contact: rafael.gabriel@salud.madrid.org)
EVA	Yes	PROG-IMT policy	Data can be shared on a scientific collaborative basis (Contact: jean-philippe.empana@inserm.fr)
HOORN	Yes	The informed consent specifically stated data will stay with the researchers of VUmc, except for scientific collaborations.	Study data are available upon request. Data requests can be sent to the Hoorn Study research group at hoornstudy@vumc.nl.
IMPROVE	Yes	There are legal (consortium agreement restrictions) and ethical (lack of patient's consensus for public data sharing) restrictions regarding public data sharing.	Data can be shared on a collaborative basis (Contact: damiano.baldassarre@unimi.it)
INVADE	Yes	There are legal restrictions due to the policy of the AOK (data owner)	Data can be shared on a scientific collaborative basis (Contact: d.sander@mac.com)
KIHD	Yes	Legal restrictions	Data can be shared on a scientific collaborative basis upon application (University of Eastern Finland, represented by the Institute of Public Health and Clinical Nutrition, contact: jussi.kauhanen@uef.fi)
Landecho et al.	No	PROG-IMT policy	Data can be shared on a scientific collaborative basis (mflandecho@unav.es)
MDCS	Yes	Swedish law regarding personal information, PROG-IMT policy	It is possible to apply for data from Malmö Diet and Cancer steering committee for scientific research (Contact: gunnar.engstrom@med.lu.se).
Niguarda-Monzino	Yes	There are legal (consortium agreement restrictions) and ethical (lack of patient's consensus for public data sharing) restrictions regarding public data sharing.	Data can be shared on a collaborative basis (Contact: damiano.baldassarre@unimi.it)
NOMAS /INVEST	Yes	Inform consents that our subjects signed restrict the sharing of individual data as well as group sharing to limited datasets with a prior approval from the respective study steering committees.	Contact NOMAS/INVEST steering committee or TRundek@med.miami.edu.
OSACA-2	No	PROG-IMT policy	Data can be shared on a scientific collaborative basis (kkitagawa@nij.twmu.ac.jp)

Study	Third party owned	Restrictions against public data sharing	Availability and contact
PIVUS	No	Swedish law regarding personal information, PROG-IMT policy	Data can be shared on a collaborative basis (lars.lind@medsci.uu.se)
PLIC	No	Legal restrictions, PROG-IMT policy	Data can be shared on a collaborative basis (alberico.catapano@unimi.it)
RIAS	No	PROG-IMT policy	Data can be shared on a scientific collaborative basis (Daniel.Staub@usb.ch)
Rotterdam	Yes	Legal restrictions prohibit sharing of data	Contact Rotterdam steering committee (Contact: o.franco@erasmusmc.nl).
SAPHIR	No	PROG-IMT policy	Data can be shared on a scientific collaborative basis (B.Iglseder@salk.at)
SHIP	Yes	Legal restrictions prohibit sharing of data	Data use, also for quality assurance issues, can be applied for via our websites (www.community-medicine.de) according to a standardized procedure.
SPARC	No	Ethical	Can be accessed upon request to David Spence orthe ethics board of Western University, London, Canada with submission of an ethics protocol (Contact: dspence@robarts.ca)
Tromsø	Yes	Legal restrictions prohibit sharing of data	Data can be shared on a scientific collaborative basis (contact steering committee or ellisiv.mathiesen@uit.no)

* For the purpose of the current analysis, the AtheroGene data were analysed by a member of the Mainz biometric team – Irene Schmidtmann – following specific statistc models provided by central statistics in Cambridge, and only the resulting estimates were delivered for pooling to central statistics. The authors of the current paper outside the Mainz team received no access privileges to the AtheroGene data.
