# Supplementary material for: Predictive value for cardiovascular events of common carotid intima media thickness and its rate of change in individuals at high cardiovascular risk – Results from the PROG-IMT collaboration
Source: PLoS One. 2018 Apr 12;13(4):e0191172. doi: 10.1371/journal.pone.0191172 (PMC5896895; doi:10.1371/journal.pone.0191172)
Supplement: S4 Table — (PDF) [file pone.0191172.s004.pdf]

List of collaborators

within the PROG-IMT study group, current from 10th June 2016

Stefan Agewall, MD, PhD, Prof.
Institute of Clinical Sciences,University of Oslo, Oslo, Norway
and
Department of Cardiology, Oslo University Hospital Ullevål, Oslo, Norway

Tadao Akizawa, MD, PhD, Prof
Division of Nephrology,Department of Medicine,Showa University School of Medicine, Tokyo, Japan

Mayuko Amaha, MD
Division of Nephrology,Department of Internal Medicine,Shinmatsudo Central General Hospital, Chiba, Japan

Mauro Amato, PhD
Centro Cardiologico Monzino, IRCCS, Milan, Italy

Steen Andersen, MD, DMSc
Steno Diabetes Center, Gentofte, Denmark

Sigmund A. Anderssen, PhD, Prof
Norwegian School of Sports Sciences, Oslo, Norway

Aleksandra Araszkiewicz, MD, PhD, Assoc. Prof.
Department of Internal Medicine and Diabetology, Poznan University of Medical Sciences, Poznan, Poland

Folkert W. Asselbergs, MD, PhD, Assoc. Prof.
Department of Cardiology,Division Heart & Lungs,University Medical Center Utrecht, Utrecht, the Netherlands

Gülay Asçi, Prof. Dr.
Nefroloji Bilim Dali,Tip Fakültesi,Ege Üniversitesi, Bornova-Izmir, Turkey

Jang-Ho Bae, MD.,PhD.,FACC.
College of Medicine, Konyang University Hospital, Daejeon, Korea
and
Heart Center, Konyang University Hospital, Daejeon, Korea

Tatyana Balakhonova, MD, PhD, Prof.
Ultrasound Vascular Laboratory,Cardiology Research Center , Moscow, Russia

Damiano Baldassarre, PhD, Prof
Centro Cardiologico Monzino, IRCCS, Milan, Italy
and
Dipartimento di Scienze Farmacologiche e Biomolecolari, Università di Milano, Milan, Italy

Edith Beishuizen
Department of General Internal Medicine, Leiden University Medical Center, Leiden, the Netherlands

Oscar Beloqui, MD, PhD
Department of Internal Medicine, University Clinic of Navarra, Navarra, Spain

Gerald Berenson, MD, Prof.
Department of Medicine, Pediatrics, Biochemistry, Epidemiology, Tulane University School of Medicine and School of Public Health and Tropical Medicine, New Orleans, USA

Göran Bergström, MD, PhD, Prof
Wallenberg Laboratory for Cardiovascular Research, Sahlgrenska Academy, Gothenburg University, Götheborg, Sweden

Enrique Bernal, MD, PhD
Infectious Diseases Unit,Hospital Reina Sofia , Murcia, Spain

Sebastjan Bevc, MD, PhD, Assist Prof
Department of Nephrology,Clinic for Internal Medicine,University Medical Centre Maribor, Maribor, Slovenia

Lokpal Bhatia, MB BCh
Faculty of Medicine,Human Development & Health Academic Unit,University of Southampton - Southampton General Hospital, Southhampton, UK
and
Southampton NIHR Biomedical Research Centre, University Hospital Southampton - Southampton General Hospital, ,

Horst Bickel, PhD
Department of Psychiatry and Psychotherapy, Technische Universität München, Munich, Germany

Stefan Blankenberg, MD, Prof.
2nd Department of Medicine, Johannes Gutenberg-Universität, Mainz, Germany
and
Department of Cardiology, University Hospital Hamburg-Eppendorf, Hamburg, Germany

Peter J. Blankestijn
Department of Nephrology, University Medical Center Utrecht, Utrecht, The Netherlands

James A Blumenthal, PhD
Department of Psychiatry and Behavioral Sciences, Duke University Medical Center, Durham, NC 27710, USA

Lena Bokemark, MD, PhD
Wallenberg Laboratory for Cardiovascular Research,Institution for Medicin, Department for Molecular and Clinical Medicine,Sahlgrenska Academy, Gothenburg University, Gothenburg, Sweden

Jackie Bosch, MSc
Population Health Research Institute, McMaster University, Hamilton, Ontario, Canada

Michiel L. Bots, MD, PhD, Prof
Julius Center for Health Sciences and Primary Care, University Medical Center Utrecht, Utrecht, the Netherlands

Frank P. Brouwers, MD, PhD
Department of Cardiology, University Medical Center Groningen, Groningen, the Netherlands

Christopher D. Byrne, MB BCh, PhD, Prof.
Faculty of Medicine,Human Development & Health Academic Unit,University of Southampton - Southampton General Hospital, Southhampton, UK
and
Southampton NIHR Biomedical Research Centre, University Hospital Southampton - Southampton General Hospital, ,

Alpaslan Bülbül, MPH
Department of Neurology, Goethe University, Frankfurt am Main, Germany

Philip C. Calder, PhD, Prof.
Faculty of Medicine,Human Development & Health Academic Unit,University of Southampton - Southampton General Hospital, Southhampton, UK
and
Southampton NIHR Biomedical Research Centre, University Hospital Southampton - Southampton General Hospital, ,

Yong-Jun Cao, MD, PhD
Department of Neurology,Institute of Neuroscience,the Second Affiliated Hospital of Soochow University, Soochow, China

Samuela Castelnuovo, PhD
Centro Dislipidemie E. Grossi Paoletti,Ospedale Ca' Granda di Niguarda , Milan, Italy

Alberico Catapano, PhD, Prof.
IRCSS Multimedica, Milan, Italy
and
Department of Pharmacological and Biomolecular Sciences, University of Milan, Milan, Italy

Chen-Huan Chen, Prof, MD
National Yang-Ming University, Taipei, Taiwan

Kuo-Liong Chien, MD, Prof
Institute of Epidemiology and Preventive Medicine,College of Public Health,National Taiwan University, Taipei, Taiwan

Ana Rosa Cunha, PhD
State University of Rio de Janeiro, Rio de Janeiro, Brazil

Francois Dabis, MD, PhD, Prof.
Centre de Recherche INSERM U.897,Institut de Santé Publique, Epidémiologie et Développement (ISPAD),Université Victor Segalen Bordeaux 2, Bordeaux Cedex, France

Jesse Dawson, Dr.
Institute of Cardiovascular and Medical Sciences,University of Glasgow, Glasgow, UK

Ralph A. DeFronzo, MD
University of Texas Health Science Center, San Antonio, USA

Jaqueline .M. Dekker, Prof
Department of Epidemiology and Biostatistics,University Medical Center , Amsterdam, the Netherlands
and
EMGO Institute for Health and Care Research, VU University Medical Center, Amsterdam, the Netherlands

Moise Desvarieux, MD, PhD, Assoc. Prof.
Department of Epidemiology,Mailman School of Public Health,Columbia University, New York, USA

Chrystosomos Dimitriadis, MD
University Department of Nephrology, Hippokration General Hospital, Thessaloniki, Greece

Pierre Ducimetiere, Prof.
University Paris_Sud Xi, Kremlin-Bicêtre, Le Kremlin-Bicêtre, France

Marcus Dörr, MD, Prof.
Department B for Internal Medicine, University Medicine Greifswald, Greifswald, Germany
and
German Center for Cardiovascular Research (DZHK),partner site Greifswald , Greifswald, Germany

Helen Eddington, MD
University Hospitals Birmingham, Birmingham, UK

Robert Ekart, MD, PhD, Assist. Prof
Department of Dialysis,Clinic for Internal Medicine,University Medical Centre Maribor, Maribor, Slovenia

Jean Philippe Empana, MD, PhD
Paris Cardiovascular Research Centre (PARCC), University Paris Descartes, Sorbonne Paris Cité, UMR-S970, Paris, France

Gunnar Engström, MD, PhD, Prof.
Department of Clinical Sciences in Malmö, Lund University, Malmö, Sweden

Mark A. Espeland, PhD, Prof.
Department of Biostatistical Sciences, Wake Forest School of Medicine, Winston-Salem, NC, USA

Christine Espinola-Klein, MD, Prof.
2nd Department of Medicine, Johannes-Gutenberg University, Mainz, Germany

Ramon Estruch, MD, PhD
Endocrinology & Nutrition Service, Hospital Clínic, Barcelona, Spain

Thorleif Etgen, MD, PD
Department of Neurology,Kliniken Südostbayern,Klinikum Traunstein, Traunstein, Germany
and
Department of Psychiatry and Psychotherapy, Technische Universität München, Munich, Germany

Marat Ezhov, MD, PhD
Atherosclerosis Department,Cardiology Research Center , Moscow, Russia

Oscar H. Franco, MD, PhD, FESC, FFPH, Prof.
Department of Epidemiology, Erasmus MC, University Medical Center Rotterdam, Rotterdam, the Netherlands

Beat Frauchiger, MD, Prof.
Department of Internal Medicine, Division of Angiology,Kantonsspital Frauenfeld, Frauenfeld, Switzerland

Alfonsa Friera, MD
Radiology Department,Hospital Universitario de la Princesa, Universidad Autónoma de Madrid, Madrid, Spain

Rafael Gabriel, Prof., MD
Escuela National de Sanidad, Instituto de Salud Carlos III, Madrid, Spain

Greg Gamble, MSc
Department of Medicine, The University of Auckland, Auckland, New Zealand

Lu Gao, MSc
MRC Biostatistics Unit,Institute of Public Health,University Forvie Site, Cambridge, UK

Rachel Georgiou
Salford Royal Hospitals NHS Foundation Trust, Salford, UK

Hertzel C. Gerstein, MD, MSc
Department of Medicine and Population Health Research Institute,McMaster University , Hamilton, Ontario, Canada

Paolo Gresele, Prof, MD, PhD
Department of Internal,Division of Internal and Cardiovascular Medicine,University of Perugia, Perugia, Italy

Liliana Grigore, MD
Centro Sisa per lo Studio della Aterosclerosi, Bassini Hospital, Cinisello Balsamo, Italy

Diederick E. Grobbee, MD, PhD, Prof
Julius Center for Health Sciences and Primary Care, University Medical Center Utrecht, Utrecht, the Netherlands

Muriel P.C. Grooteman
Institute for Cardiovascular Research VU Medical Center (ICaR-VU),VU Medical Center, Amsterdam, The Netherlands
and
Department of Nephrology, VU Medical Center, Amsterdam, The Netherlands

Giuseppe Guglielmini, MD, PhD
Division of Internal and Cardiovascular Medicine,Department of Medicine,University of Perugia, Perugia, Italy

Félix Gutiérrez, MD, PhD
Clinical Medicine,Hospital General Universitario de Elche,University Miguel Hernández, Elche, Alicante, Spain

Markolf Hanefeld, Prof
Study Centre Professor Hanefeld, GWT,TU Dresden, Dresden, Germany

Apostolos I. Hatzitolios, MD, PhD, FESH, Prof.
Head of first1 Propedeutic Department of Internal Medicine,Medical School,Aristotle University of Thessaloniki - AHEPA Hospital, Thessaloniki, Greece

Bo Hedblad, MD, PhD, Prof
Department of Clinical Sciences in Malmö, Lund University, Malmö, Sweden

Frans A. Helmond, PhD
Merck Research Laboratories, Kenilworth, NJ, USA

Loghman Henareh, M.D., Ph.D., Prof.
Department of Medicine, Huddinge (MedH), H7,Karolinska Institutet , Stockholm, Sweden

Peter Higgins, Dr.
Institute of Cardiovascular and Medical Sciences,University of Glasgow, Glasgow, UK

Alan Hinderliter, MD
Department of Medicine, University of North Carolina, Chapel Hill, USA

Albert Hofman, MD, PhD, Prof.
Department of Epidemiology, Erasmus University Medical Center, Rotterdam, the Netherlands

Radovan Hojs, MD, PhD, Prof
Department of Nephrology,Clinic for Internal Medicine,University Medical Centre Maribor, Maribor, Slovenia
and
Faculty of Medicine, University of Maribor, Maribor, Slovenia

Hirokazu Honda, MD, PhD, Assoc. Prof.
Showa University Koto Toyosu Hospital, Division of Nephrology, Department of Medicine
Tokyo, Japan

Satoshi Hoshide, MD
Department of Medicine,Division of Cardiovascular Medicine,Jichi Medical University School of Medicine, Tochigi, Japan

Menno V. Huisman
Department of Thrombosis and Hemostasis, Leiden University Medical Center, Leiden, the Netherlands

Joseph Hung, Winthrop Professor, MBBS (hons) FRACP FACC
Department of Cardiovascular Medicine, Sir Charles Gairdner Hospital, Nedlands, Australia
and
School of Medicine and Pharmacology, University of Western Australia, Nedlands, Australia

Bernhard Iglseder, MD, Prof
Parcelsus Medical University, Salzburg, Austria
and
Department of Geriatric Medicine, Gemeinnützige Salzburger Landeskliniken Betriebsgesellschaft GmbH Christian-Doppler-Klinik, Salzburg, Austria

M. Arfan Ikram, MD, PhD, Assoc. Prof.
Department of Epidemiology, Erasmus University Medical Center, Rotterdam, the Netherlands
and
Department of Neurology, Erasmus University Medical Center, Rotterdam, the Netherlands
and
Department of Radiology, Erasmus University Medical Center, Rotterdam, the Netherlands

Raffaele Izzo, MD, Prof.
School of Medicine, Federico II University, Naples, Italy

Lisa M Jamieson, Assoc Prof
Australian Research Centre for Population Oral Health,School of Dentistry,The Univeristy of Adelaide, Adelaide, Australia

Tomas Jogestrand, M.D., Ph.D., Prof.
Department of Laboratory Medicine (LABMED), H5, Division of clinical physiology,Karolinska Universitetssjukhuset, Huddinge , Stockholm, Sweden

Stein Harald Johnsen, MD, PhD, Assoc. Prof.
Department of Clinical Medicine, University of Tromsø, Tromsø, Norway
and
Department of Neurology, University Hospital of Northern Norway, Tromsø, Norway

Aleksandar Jovanovic, MD, PhD, Prof
Faculty of Medicine, University of Prishtina, Prishtina\Kosovska Mitrovica, Serbia

Anna Kablak-Ziembicka, MD, PhD, Prof.
Department of Interventional Cardiology,Institute of Cardiology,Collegium Medicum Jagiellonian University, Krakow, Poland

Philip Kalra, MD, PhD, Prof.
Department of Renal Medicine,Manchester Academic Health Sciences Centre,Salford Royal Hospital, Manchester, UK

Kostas Kapellas
Australian Research Centre for Population Oral Health,School of Dentistry,The University of Adelaide, Adelaide, Australia

Kazuomi Kario
Department of Medicine,Division of Cardiovascular Medicine,Jichi Medical University School of Medicine, Tochigi, Japan

John JP Kastelein, MD, Prof
Department of Vascular Medicine,Academic Medical Center,University of Amsterdam, Amsterdam, the Netherlands

Akihiko Kato, MD, Prof.
Division of Blood Purification, Hamamatsu University Hospital, Hamamatsu, Japan

Jussi Kauhanen, MD, Prof
Institute of Public Health and Clinical Nutrition, University of Eastern Finland, Kuopio Campus, Kuopio, Finland

Maryam Kavousi, MD, PhD
Department of Epidemiology and Biostatistics, Erasmus Medical Center, Rotterdam, the Netherlands

Masanori Kawasaki, MD, PhD, Assoc. Prof
Department of Cardiology, Gifu University Graduate School of Medicine, Gifu, Japan

Kerstin Kempf, PhD
Düsseldorf Catholic Hospital Group (VKKD),West-German Centre of Diabetes and Health (WDGZ) , Düsseldorf, Germany

Stefan Kiechl, MD, Prof
Department of Neurology, Medical University Innsbruck, Innsbruck, Austria

Jang-Young Kim, MD PhD
Department of Cardiology,Institute of Genomic Cohort,College of Medicine Yonsei University, Wonju, Korea

Kazuo Kitagawa, MD, PhD
Department of Neurology, Tokyo Women's Medical University, Tokyo, Japan

Sverre E. Kjeldsen, MD, PhD, Prof.
Department of Cardiology, Ullevaal University Hospital, Oslo, Norway

Sang Back Koh, MD PhD
Preventive Medicine, College of Medicine,Institute of Genomic Cohort,Yonsei University, Wonju, Korea

Svetlana Kostic, MSc, MD
Primarius Institute for Therapy and Rehabilitation,"Niska Banja" , Nis, Serbia

Manuel F Landecho, MD, PhD
Department of Internal Medicine, University Clinic of Navarra, Navarra, Spain

Tatjana Lazarevic, MA
Faculty of Medicine, University of Kragujevac, Kragujevac, Serbia

Moo-Sik Lee, MD., PhD., Prof.
College of Medicine, Konyang University Hospital, Daejeon, Korea
and
Department of Preventive Medicine, Konyang University, Daejeon, Korea

Seung Hwan Lee, M.D., PhD, Prof.
Department of Cardiology,College of Medicine,Yonsei University, Wonju, Korea

Wattana Leowattana, Assoc. Prof.
Department of Clinical Tropical Medicine,Faculty of Tropical Medicine,Mahidol University, Rachatawee, Bangkok, Thailand

Ximing Liao, BSc, MSc, PhD
Department of Neurology, Goethe University, Frankfurt am Main, Germany

Hung-Ju Lin, MD
Department of Internal Medicine, National Taiwan University Hospital, Taipei, Taiwan

Yao-Ping Lin, MD
Taipei Veterans General Hospital, Taipei, Taiwan

Lars Lind, MD, PhD, Prof
Department of Medicine, Uppsala University, Uppsala, Sweden

Chun-Feng Liu, MD, PhD, Prof
Department of Neurology,Institute of Neuroscience,the Second Affiliated Affiliated Hospital of Soochow University, Soochow, China

Jing Liu, MD, PhD, Prof.
Department of Epidemiology,Beijing Institute of Heart, Lung and Blood Vessel Diseases,Beijing Anzhen Hospital, Capital Medical University, Beijing, China

Eva Lonn, MD, MSc, FRCPC, FACC, Prof.
Department of Medicine and Population Health Research Institute, McMaster University, Hamilton, Ontario, Canada

Matthias W. Lorenz, MD, PD
Department of Neurology, Goethe University, Frankfurt am Main, Germany

Dongmei Ma, MSc
Institute of Child and Adolescent Health, School of Public Health,Peking University, Beijing, China

Dianna Magliano, Assoc. Prof
BakerIDI Heart and Diabetes Institute , Melbourne, Australia

Stephan Martin, Prof. Dr.
Düsseldorf Catholic Hospital Group (VKKD),West-German Centre of Diabetes and Health (WDGZ) , Düsseldorf, Germany

Mar Masiá, MD, PhD
Cinical Medicine,Hospital General Universitario de Elche,University Miguel Hernández, Elche, Alicante, Spain

Ellisiv B. Mathiesen, MD, PhD, Prof.
Department of Clinical Medicine, UiT The Arctic University of Norway, Tromsø, Norway

Wolfgang Mayer-Berger
Centre for Cardiovascular Rehabilitation, Leichlingen, Germany

Barry P. McGrath, Prof
Department of Vascular Sciences, Monash University, Dandenong Hospital, Melbourne, Australia

Stela McLachlan, PhD
Centre for Population Health Sciences,Usher Institute of Population Health Sciences and Informatics,University of Edinburgh, Edinburgh, UK

John McNeil, PhD, Prof., MBBS
School of Public Health and Preventive Medicine, Monash University, Melbourne, Australia

Brendan McQuillan, Assoc. Prof, MBBS PhD FRACP
Department of Cardiovascular Medicine, Sir Charles Gairdner Hospital, Nedlands, Australia
and
School of Medicine and Pharmacology, University of Western Australia, Nedlands, Australia

Patrick Mercié, Prof
Service de Médecine Interne,Hôpital Saint-André,CHU de Bordeaux, Bordeaux, France

Rino Migliacci, MD
Division of Internal Medicine,Ospedale della Valdichiana"S.Margherita" , Cortona, Italy

Dragan Mijalkovic, MD
Primarius,Polyclinic "Kardiomedika" , Nis, Serbia

Firouzeh Moeinzadeh, MD
Isfahan Kidney Disease Research Center,Isfahan University of Medical Sciences, Isfahan, Iran

Mojgan Mortazavi, MD, Assoc. Prof.
Nephrology departement,Isfahan Kidney Diseases Research Center,Isfahan University of medical sciences, Isfahan, Iran

Titus Francis Msoka, MD, PhD
Kilimanjaro Christian Medical Centre (KCMC) , Moshi, Tanzania
and
Kilimanjaro Clinical Research Institute (KCRI) , Moshi, Tanzania

Veronica A. Myasoedova, MD, PhD
Laboratory of Angiopathology,Institute of General Pathology and Pathophysiology , Moscow, Russia

Michiaki Nagai, MD
Department of Medicine,Division of Cardiovascular Medicine,Jichi Medical University School of Medicine, Tochigi, Japan

Tsukasa Nakamura, MD, PhD
Division of Nephrology,Department of Internal Medicine,Shinmatsudo Central General Hospital, Chiba, Japan

Prabath W.B. Nanayakkara
Department of Clinical Neurophysiology,Medical Center,VU University Amsterdam, Amsterdam, the Netherlands

Dariusz Naskret, MD, PhD
Department of Internal Medicine and Diabetology, Poznan University of Medical Sciences, Poznan, Poland

Mario Fritsch Neves, MD, PhD
State University of Rio de Janeiro, Rio de Janeiro, Brazil

Pythia T. Nieuwkerk, PhD
Department of Medical Psychology, Academic Medical Center, Amsterdam, the Netherlands

Giel Nijpels, MD, PhD
Department of General Practice,VU University Medical Center , Amsterdam, the Netherlands
and
EMGO Institute for Health and Care Research, VU University Medical Center, Amsterdam, the Netherlands

Giuseppe D. Norata, PhD
Dipartimento di Scienze Farmacologiche e Biomolecolari, Università degli Studi di Milano, Milan, Italy
and
SISA Center for the Study of Atherosclerosis, Bassini Hospital, Cinisello Balsamo, Italy

George Ntaios, MD, MSc (Stroke Med), PhD, FESO
Department of Medicine, University of Thessaly, Larissa, Greece

Shuhei Okazaki, MD
Department of Neurology, Osaka University Graduate School of Medicine, Osaka, Japan

Michael Hecht Olsen, MD, PhD, DMSc, Prof
Cardiovascular and Metabolic Preventive Clinic,Department of Endocrinology,Odense University Hospital, Odense, Denmark

Alexander N. Orekhov, PhD, DSc
Institute for atherosclerosis Research,Skolkovo Innovation Center, Moscow, Russia
and
Laboratory of Angiopathology,Institute of General Pathology and Pathophysiology , Moscow, Russia

Aikaterini Papagianni, MD, Assoc. Prof.
University Department of Nephrology, Hippokration General Hospital, Thessaloniki, Greece

Hyun Woong Park, M.D.
College of Medicine, Konyang University Hospital, Daejeon, Korea

Grace Parraga, Dr
Robarts Research Institute,Western University, London, Canada

Sharif Pasha
Department of General Internal Medicine, Leiden University Medical Center, Leiden, the Netherlands

Matthieu Plichart, MD, PhD
Assistance Publique, Hôpitaux de Paris, Hôpital Broca, Paris, France
and
Paris Cardiovascular Research Centre (PARCC), University Paris Descartes, Sorbonne Paris Cité, UMR-S970, Paris, France

Janice Pogue, PhD
Population Health Research Institute, McMaster University, Hamilton, Ontario, Canada

Joseph F. Polak, MD, MPH, Prof
Tufts University School of Medicine, Tufts Medical Center, Boston, USA

Holger Poppert, MD, PhD
Department of Neurology, Technische Universität München, Munich, Germany

David Preiss, MD, PhD
BHF Glasgow Cardiovascular Research Centre, University of Glasgow, Glasgow, UK

Jackie F. Price, MD
Usher Institute of Population Health Sciences and Informatics,University of Edinburgh, Edinburgh, UK

Tadeusz Przewlocki, MD, PhD, Prof.
Department of Interventional Cardiology,Institute of Cardiology,Collegium Medicum Jagiellonian University, Krakow, Poland

Joel Raichlen, MD
AstraZeneca, Wilmongton, DE, USA

Peter Reaven, MD, Prof.
Dept. of Medicine,Phoenix VA Health Care System,University of Arizona, Phoenix, USA

Peter Reiss, MD, PhD, Prof.
Amsterdam Institute for Global Health and Development, University of Amsterdam, Amsterdam, the Netherlands
and
Department of Global Health, Academic Medical Center, Amsterdam, the Netherlands

Christine Robertson, MBChB
Centre for Population Health Sciences,Usher Institute of Population Health Sciences and Informatics,University of Edinburgh, Edinburgh, UK

Kimmo Ronkainen, MSc
Institute of Public Health and Clinical Nutrition, University of Eastern Finland, Kuopio Campus, Kuopio, Finland

Emilio Ros, MD, PhD
Endocrinology & Nutrition Service, Hospital Clínic, Barcelona, Spain

Signe Rosenlund, MD
Steno Diabetes Center , Gentofte, Denmark

Peter Rossing, MD, DMSc, Prof.
Steno Diabetes Center , Gentofte, Denmark

Maria Rosvall, MD, PhD, Assoc. Prof.
Department of Clinicla Sciences in Malmö, Lund University, Malmö, Sweden

Francesco Rozza, MD, Prof.
Department of Medicine and Surgery, University of Salerno, Salerno, Italy

Tatjana Rundek, MD, PhD, Prof.
Department of Neurology,Miller School of Medicine,University of Miami, Miami, USA

Mohammad Saadatnia, MD, Assoc. Prof.
Al-Zahra Hospital, Isfahan University of Medical Sciences, Isfahan, Iran

Ralph L. Sacco, MD, MS, Prof.
Department of Neurology,Miller School of Medicine,University of Miami, Miami, USA

Maya Safarova, M.D.
Atherosclerosis Department,Cardiology Research Center , Moscow, Russia

Dirk Sander, MD, Prof
Department of Neurology, Benedictus Hospital Tutzing & Feldafing, Feldafing, Germany
and
Department of Neurology, Technische Universität München, Munich, Germany

Kerstin Sander, MD, PD
Department of Psychosomatic,Schön Klinik , Berchtesgadener Land, Germany

Eiichi Sato, MD
Division of Nephrology,Department of Internal Medicine , Chiba, Japan

Naveed Sattar, MD, PhD, Prof
BHF Glasgow Cardiovascular Research Centre, University of Glasgow, Glasgow, UK

Christos Savopoulos, MD, PhD, Assoc. Prof
1st Propedeutic Department of Internal Medicine, Aristotles University of Thessaloniki, Thessaloniki, Greece

Frank Scheckenbach, MSc, PhD
Department of Neurology, Goethe University, Frankfurt am Main, Germany

Caroline Schmidt, PhD, Assoc. Prof.
Walleng Laboratory for Cardiovascular Research, University of Gothenburg, Gothenburg, Sweden

Irene Schmidtmann, Dr.
Institut fuer Medizinische Biometrie, Epidemiologie und Informatik (IMBEI), Universitaetsmedizin Mainz, Mainz, Germany

Ulf Schminke, MD, Prof
Department of Neurology, Greifswald University Clinic, Greifswald, Germany

Michael Schneider, Dr.
Dept HR Services & Expertise Center,Boehringer Ingelheim Pharma GmbH & Co. KG , Ingelheim am Rhein, Germany
and
Mannheim Institute for Public Health,Medical Faculty Mannheim,Ruprecht-Karls University Heidelberg, Mannheim, Germany

Ercan Sevinc Ok, MD
Division of Nephrology, Izmir Bozyaka Education and Research Hospital, Izmir, Turkey

Norman Sharpe, MD, Prof.
Heart Foundation, Ellerslie, Auckland, New Zealand

Patrick Sheridan, MSc
Population Health Research Institute, McMaster University, Hamilton, Ontario, Canada

Andrew Sherwood, PhD
Department of Psychiatry and Behavioral Sciences, Duke University Medical Center, Durham, NC 27710, USA

Cesare R. Sirtori, MD, PhD, Prof.
Center of Dyslipidemias,Niguarda Ca' Granda Hospital, Milano, Italy

Matthias Sitzer, MD, Prof.
Department of Neuology, Klinikum Herford, Herford, Germany
and
Department of Neurology, Goethe University, Frankfurt am Main, Germany

Michael Skilton, PhD
Boden Institute of Obesity, Nutrition, Exercise and Eating Disorders, University of Sydney, Sydney, Australia

Patrick J Smith, PhD, MPH
Department of Psychiatry and Behavioral Sciences, Duke University Medical Center, Durham, NC 27710, USA

Igor A. Sobenin, MD, PhD, DSc
Laboratory of Angiopathology,Institute of General Pathology and Pathophysiology , Moscow, Russia
and
Laboratory of Medical Genetics, Department of Cardiovascular Pathology,AM Myasnikov Institute of Clinical Cardiology,Russian Cardiology Research and Production Complex, Moscow, Russia

J. David Spence, MD, FRCPC, FAHA
Stroke Prevention & Atherosclerosis Research Centre,Robarts Research Institute,Western University, London, Canada

Sathanur R. Srinivasan, PhD, Prof.
Center for Cardiovascular Health, Department of Epidemiology, Biochemistry, Tulane University School of Public Health and Tropical Medicine, New Orleans, USA

Daniel Staub, MD, Prof.
Department of Angiology, University Hospital Basel, Basel, Switzerland

CDA Stehouwer, MD, PhD, FESC
Department of Internal Medicine and Cardiovascular Research Institute Maastricht (CARIM), Maastricht University Medical Centre, Maastricht, the Netherlands

Helmuth Steinmetz, MD, Prof
Department of Neurology, Goethe University, Frankfurt am Main, Germany

Radojica Stolic, MD, PhD, Prof
Faculty of Medicine, University of Kragujevac, Kragujevac, Serbia

Erik Stroes, MD, PhD
Department of Vascular Medicine, Academic Medical Center, Amsterdam, the Netherlands

Ta-Chen Su, MD, PhD, Assoc. Prof.
Department of Internal Medicine, National Taiwan University Hospital, Taipei, Taiwan

Carmen Suarez, MD, PhD
Internal Medicine Department,Hospital Universitario de la Princesa,Universidad Autónoma de Madrid, Madrid, Spain

Ivan S. Tasic, MD, PhD, Prof.
Faculty of Medicine,Institute for Therapy and Rehabilitation,University of Nis, "Niska Banja", Serbia

Rodolphe Thiébaut, Dr.
Centre de Recherche INSERM U.897,Institut de Santé Publique, Epidémiologie et Développement (ISPAD),Université Victor Segalen Bordeaux 2, Bordeaux Cedex, France

Peter L. Thompson, Clinical Professor, MD, FRACP, FACC, MBA
Heart Research Institute of WA and Department of Cardiovascular Medicine, Sir Charles Gairdner Hospital, Nedlands, Australia

Simon G. Thompson, DSc, Prof.
Department of Public Health and Primary Care,School of Clinical Medicine,University of Cambridge, Cambridge, UK

Estefania Toledo, MD, MPH, PhD
Centro de Investigación Biomédica en Red-Fisiopatología de la Obesidad y la Nutrición (CIBERobn), , Spain
and
Department of Preventive Medicine and Public Health, University of Navarra, Pamplona, Spain

Elena Tremoli, PhD, Prof
Centro Cardiologico Monzino, IRCCS, Milan, Italy
and
Dipartimento di Scienze Farmacologiche e Biomolecolari, Università di Milano, Milan, Italy

Devjit Tripathy, MD, PhD
University of Texas Health Science Center, San Antonio, USA

Tomi-Pekka Tuomainen, MD, PhD, Prof
Institute of Public Health and Clinical Nutrition, University of Eastern Finland, Kuopio Campus, Kuopio, Finland

Aleksandra Uruska, MD, PhD
Department of Internal Medicine and Diabetology, Poznan University of Medical Sciences, Poznan, Poland

Heiko Uthoff, MD
Department of Angiology, University Hospital Basel, Basel, Switzerland

Fabrizio Veglia, PhD
Centro Cardiologico Monzino, IRCCS, Milan, Italy

Frank L.J. Visseren, MD, Prof
Department of Vascular Medicine, University Medical Centre Utrecht, Utrecht, the Netherlands

Henry Völzke, MD, Prof
German Center for Cardiovascular Research (DZHK),partner site Greifswald , Greifswald, Germany
and
Institute for Community Medicine, SHIP/Clinical-Epidemiological Research, Greifswald, Germany

Kristian Wachtell, MD, PhD, Assoc. Prof.
Department of Cardiology, Gentofte University Hospital, Copenhagen, Denmark

Matthew Walters, Prof.
Institute of Cardiovascular and Medical Sciences,University of Glasgow, Glasgow, UK

Zhenghe Wang, MD
Institute of Child and Adolescent Health, School of Public Health,Peking University, Beijing, China

Thapat Wannarong, MD
Stroke Prevention & Atherosclerosis Research Centre,Robarts Research Institute,Western University, London, Canada
and
Department of Internal Medicine, Faculty of Medicine Siriraj Hospital, Mahidol University, Bangkok, Thailand

Gillian Whalley, PhD, Prof.
Faculty of Social and Health Sciences, Unitec, Auckland, New Zealand

Johann Willeit, MD, Prof.
Department of Neurology, Medical University Innsbruck, Innsbruck, Austria

Peter Willeit, PhD
Department of Neurology, Medical University Innsbruck, Innsbruck, Austria
and
Department of Public Health and Primary Care,School of Clinical Medicine,University of Cambridge, Cambridge, UK

Miles D. Witham, Dr.
Ninewells Hospital, Ageing and Health Ninewells Hospital,Ninewells Hospital & Medical School, Dundee, UK

Wuxiang Xie, MD, PhD, Assist. Prof.
Department of Epidemiology, Beijing Institute of Heart, Lung and Blood Vessel Diseases,Beijing Anzhen Hospital, Capital Medical University, Beijing, China

Kiyofumi Yamada, MD, PhD
Departments of Neurosurgery, Gifu University Graduate School of Medicine, Gifu, Japan

David N. Yanez, PhD, Assoc. Prof
Department of Biostatistics, University of Washington, Seattle, USA

Shinichi Yoshimura, MD, PhD, Assoc. Prof.
Departments of Neurosurgery, Gifu University Graduate School of Medicine, Gifu, Japan

Wen-Chung Yu, MD; Assoc. Prof
National Yang-Ming University, Taipei, Taiwan
and
Division of Cardiology, Taipei Veterans General Hospital, Taipei, Taiwan

Salim Yusuf, MD, Dphil
Department of Medicine and Population Health Research Institute, McMaster University, Hamilton, Ontario, Canada

Dong Zhao, MD, PhD, Prof.
Department of Epidemiology,Beijing Institute of Heart, Lung and Blood Vessel Diseases,Beijing Anzhen Hospital, Capital Medical University, Beijing, China

Zhi-Yong Zou
Institute of Child and Adolescent Health, School of Public Health,Peking University, Beijing, China

Sophia Zoungas, Assoc. Prof.
School of Public Health and Preventive Medicine, Monash University, Melbourne, Australia

Dorota A. Zozulinska-Ziólkiewicz, MD, PhD, Prof.
Department of Internal Medicine and Diabetology, Poznan University of Medical Sciences, Poznan, Poland

Eric de Groot, MD, PhD
Imagelabonline & Cardiovascular,Eindhoven and Clinical Epidemiology and Biostatistics,Academic Medical Centre, Amsterdam, the Netherlands

Nicola de Luca, MD, Prof.
School of Medicine, Federico II University, Naples, Italy

Pieter M. ter Wee, Prof Dr.
Department of Nephrology,Medical Center,VU University Amsterdam, Amsterdam, the Netherlands

Michiel A. van Agtmael, MD, PhD
Department of Internal Medicine, VU University Medical Center, Amsterdam, the Netherlands

Wiek van Gilst, PhD, Prof
Department of Experimental Cardiology, University Medical Center Groningen, Groningen, the Netherlands

Marit G. A. van Vonderen, MD, PhD
Medical Center Leeuwarden, Leeuwarden, the Netherlands

Statistical Advisor:
Simon Thompson, DSc, Prof
Department of Public Health and Primary Care, School of Clinical Medicine,University of Cambridge, Cambridge, UK

Principal Investigator:
Matthias W. Lorenz, MD, Prof.
Department of Neurology, Goethe University, Frankfurt am Main, Germany
