# Supplementary material for: Predictive value for cardiovascular events of common carotid intima media thickness and its rate of change in individuals at high cardiovascular risk – Results from the PROG-IMT collaboration
Source: PLoS One. 2018 Apr 12;13(4):e0191172. doi: 10.1371/journal.pone.0191172 (PMC5896895; doi:10.1371/journal.pone.0191172)
Supplement: S1 Fig — (DOCX) [file pone.0191172.s005.docx]

S1 Fig: Flowchart on available studies

2513 publications screened

on the basis of publicly available information

16 studies

dit not respond to multiple screening contacts

513 studies

known to be ineligible

610 studies potentially eligible

60 cohorts

known to be eligible

18 study teams declined collaboration

9 study teams

failed to deliver data in time

42 study teams assured collaboration

2 cohorts

could not be used as no endpoints remained after exclusions

33 cohorts

delivered data

31 cohorts

used in this report
