# Supplementary material for: Predictive value for cardiovascular events of common carotid intima media thickness and its rate of change in individuals at high cardiovascular risk – Results from the PROG-IMT collaboration
Source: PLoS One. 2018 Apr 12;13(4):e0191172. doi: 10.1371/journal.pone.0191172 (PMC5896895; doi:10.1371/journal.pone.0191172)
Supplement: S2 Fig — Left panel: HR for MI per one SD of annual mean CCA-IMT change, adjusted for age, sex and average mean CCA-IMT (model 1). Right panel: HR for MI per one SD of average mean CCA-IMT, adjusted for age, sex and annual mean CCA-IMT change (model 1). (DOCX) [file pone.0191172.s006.docx]

S2 Fig: Forest plots of the HR of MI in group A (asymptomatic individuals with three or more CVD risk factors) with 95% CIs


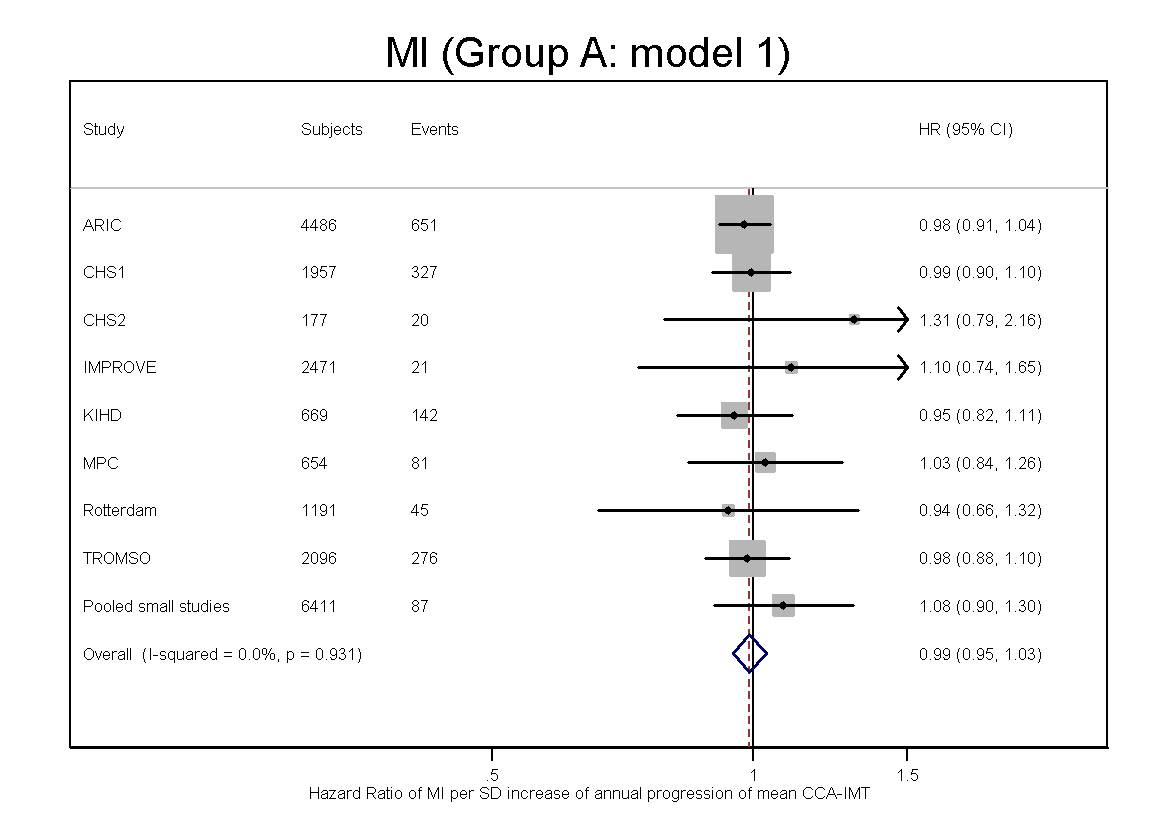

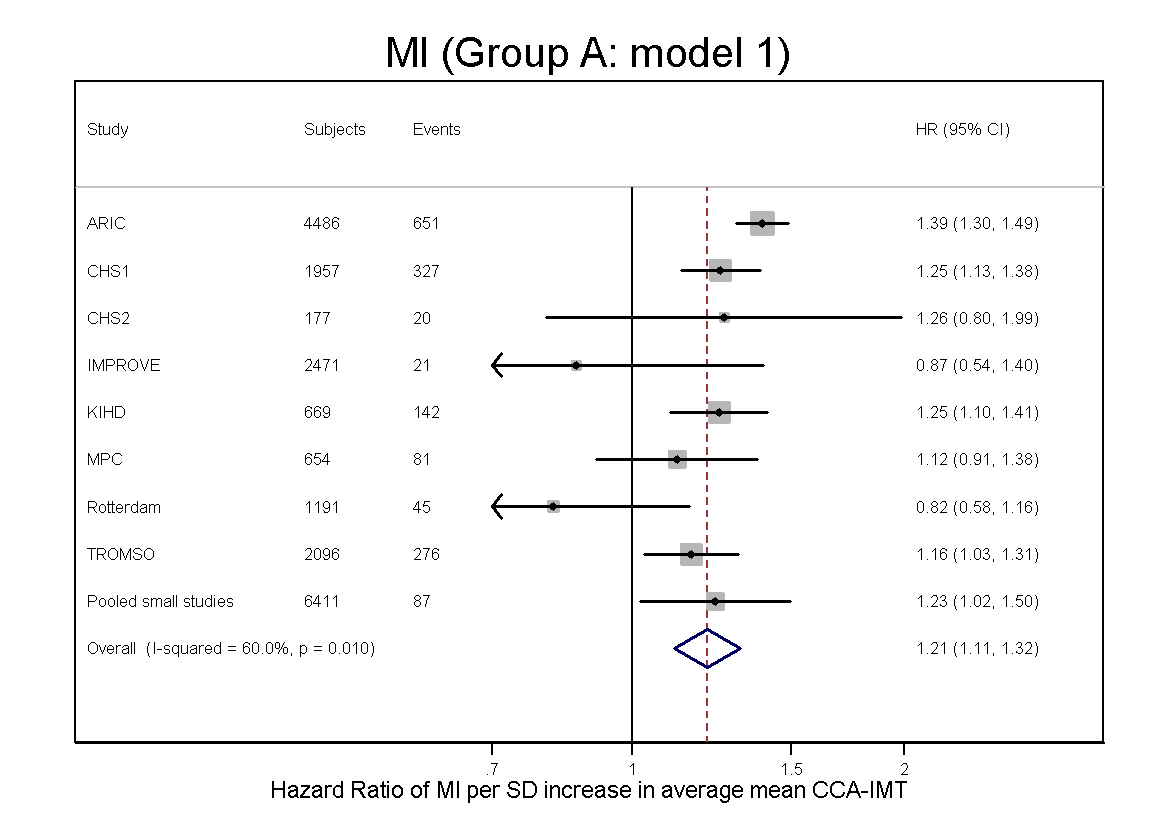


Left panel: HR for MI per one SD of annual mean CCA-IMT change, adjusted for age, sex and average mean CCA-IMT (model 1)

Right panel: HR for MI per one SD of average mean CCA-IMT, adjusted for age, sex and annual mean CCA-IMT change (model 1)
