# Supplementary material for: Predictive value for cardiovascular events of common carotid intima media thickness and its rate of change in individuals at high cardiovascular risk – Results from the PROG-IMT collaboration
Source: PLoS One. 2018 Apr 12;13(4):e0191172. doi: 10.1371/journal.pone.0191172 (PMC5896895; doi:10.1371/journal.pone.0191172)
Supplement: S6 Fig — The size of each circle represents the precision of the log HR. Left panel: Model 1 (HR adjusted for age, sex, and average mean CCA-IMT): weighted regression line y = 7.07+0.004*x (p = 0.34). Right panel: Model 2 (HR adjusted for age, sex, average mean CCA-IMT and other CVD risk factors): weighted regression line y = -9.43+0.005*x (p = 0.32). (DOCX) [file pone.0191172.s010.docx]

S6 Fig: Meta-regression plots for the HR (combined endpoint) per SD of annual mean CCA-IMT change, by the year of the study start for group A cohorts. The size of each circle represents the precision of the log HR.


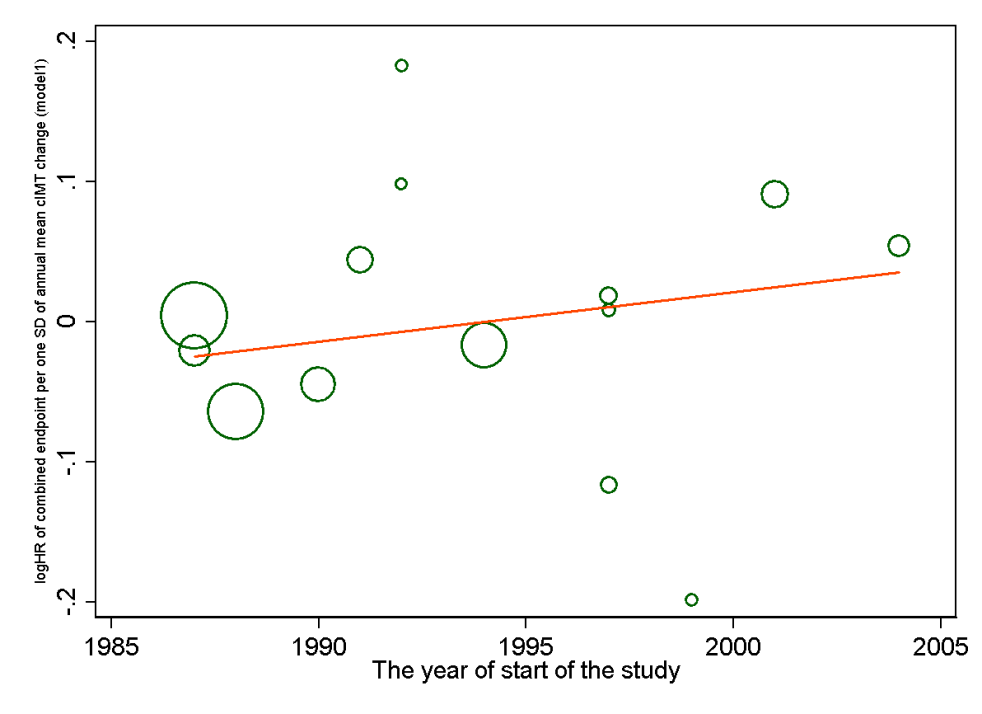

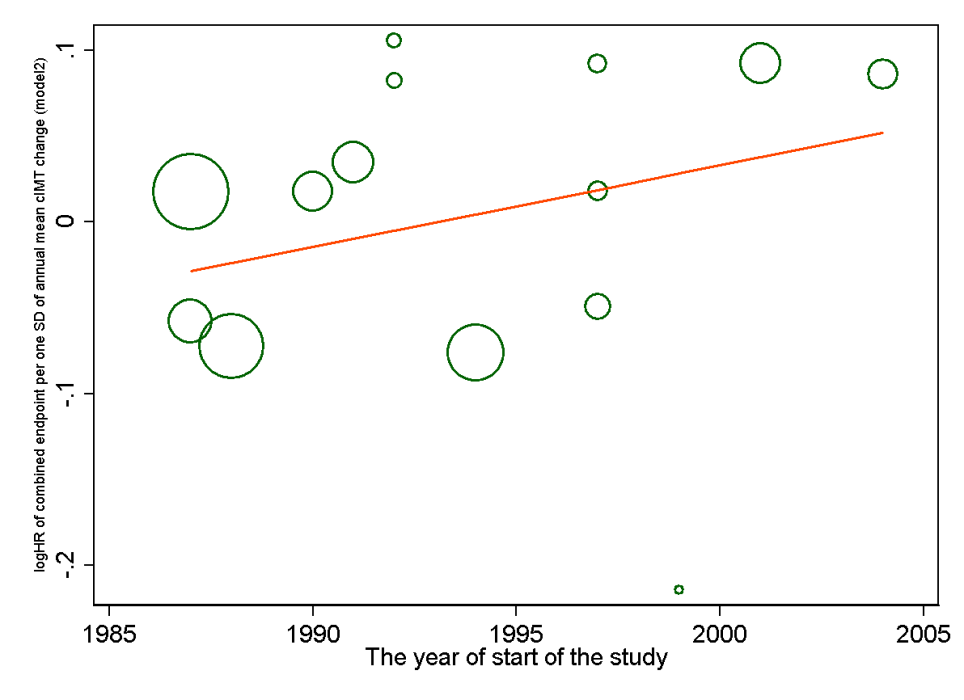


Left panel: Model 1 (HR adjusted for age, sex, and average mean CCA-IMT): weighted regression line y=7.07+0.004*x (p=0.34)

Right panel: Model 2 (HR adjusted for age, sex, average mean CCA-IMT and other CVD risk factors): weighted regression line y=-9.43+0.005*x (p=0.32)
