# Supplementary material for: Predictive value for cardiovascular events of common carotid intima media thickness and its rate of change in individuals at high cardiovascular risk – Results from the PROG-IMT collaboration
Source: PLoS One. 2018 Apr 12;13(4):e0191172. doi: 10.1371/journal.pone.0191172 (PMC5896895; doi:10.1371/journal.pone.0191172)
Supplement: S7 Fig — Group B (asymptomatic individuals with carotid plaques), HR adjusted for age, sex and average mean CCA-IMT (model 1). (DOCX) [file pone.0191172.s011.docx]

S7 Fig: Forest plots of the HR of the combined endpoint per one SD of annual mean CCA-IMT change, grouped by IMT measurement protocol, with 95% CIs


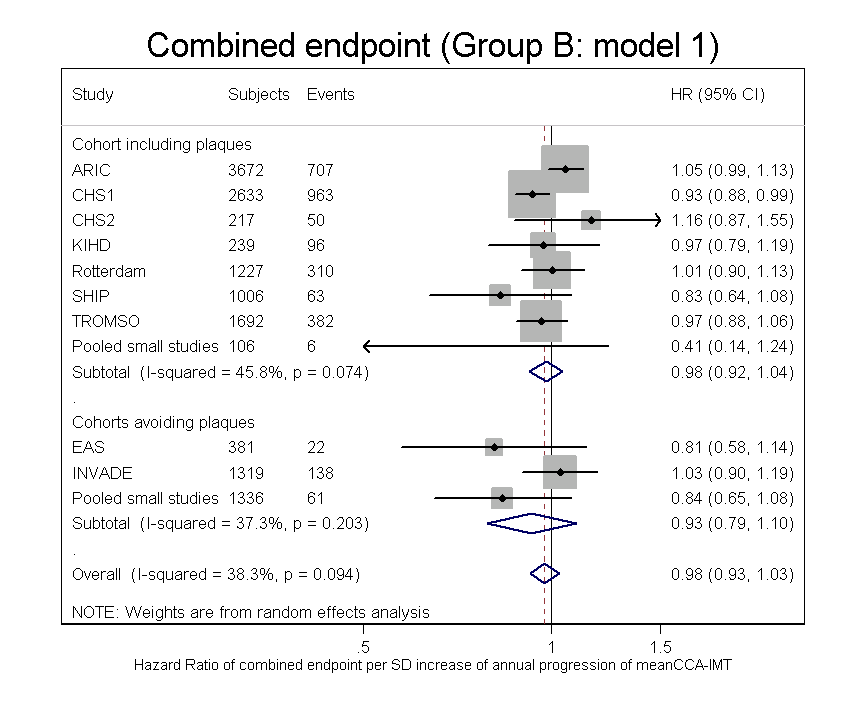


Group B (asymptomatic individuals with carotid plaques), HR adjusted for age, sex and average mean CCA-IMT (model 1)
